# Hornerin mediates phosphorylation of the polo-box domain in Plk1 by Chk1 to induce death in mitosis

**DOI:** 10.1038/s41418-023-01208-y

**Published:** 2023-08-18

**Authors:** Haiyu Song, Eun Ho Kim, Jihee Hong, Dasom Gwon, Jee Won Kim, Gyu-Un Bae, Chang-Young Jang

**Affiliations:** 1 Research Institute of Pharmaceutical Sciences, College of Pharmacy, Sookmyung Women’s University, Seoul, 04310 Republic of Korea; 2 Department of Biochemistry, School of Medicine, Catholic University of Daegu, Daegu, 42472 Republic of Korea

**Keywords:** Cell biology, Cancer

## Abstract

The centrosome assembles a bipolar spindle for faithful chromosome segregation during mitosis. To prevent the inheritance of DNA damage, the DNA damage response (DDR) triggers programmed spindle multipolarity and concomitant death in mitosis through a poorly understood mechanism. We identified hornerin, which forms a complex with checkpoint kinase 1 (Chk1) and polo-like kinase 1 (Plk1) to mediate phosphorylation at the polo-box domain (PBD) of Plk1, as the link between the DDR and death in mitosis. We demonstrate that hornerin mediates DDR-induced precocious centriole disengagement through a dichotomous mechanism that includes sequestration of Sgo1 and Plk1 in the cytoplasm through phosphorylation of the PBD in Plk1 by Chk1. Phosphorylation of the PBD in Plk1 abolishes the interaction with Sgo1 and phosphorylation-dependent Sgo1 translocation to the centrosome, leading to precocious centriole disengagement and spindle multipolarity. Mechanistically, hornerin traps phosphorylated Plk1 in the cytoplasm. Furthermore, PBD phosphorylation inactivates Plk1 and disrupts Cep192::Aurora A::Plk1 complex translocation to the centrosome and concurrent centrosome maturation. Remarkably, hornerin depletion leads to chemoresistance against DNA damaging agents by attenuating DDR-induced death in mitosis. These results reveal how the DDR eradicates mitotic cells harboring DNA damage to ensure genome integrity during cell division.

## Introduction

The DNA damage response (DDR) transmits signals to cell cycle checkpoints and coordinates DNA repair and apoptosis to prevent the generation of deleterious mutations and carcinogenesis [1, 2]. Of note, the core DNA repair machinery is suppressed during mitosis due to the fusogenic potential of mitotic telomeres [3]. Despite the effectiveness of cell cycle checkpoints in safeguarding genomic integrity, excessive DNA damage or weakened G2/M checkpoint probably results in premature or inappropriate onset of mitosis and concomitant mitotic derangement, which are resolved by delayed mitosis-associated cell death, mitotic catastrophe, during mitosis or following G1 [4–6]. Although the DDR produces binuclear cells through the ATR-Chk1-Aurora B pathway and concomitant cell death or senescence in the next G1 phase [7], the intricate mechanism of DDR-mediated death in mitosis (DiM), which is an indispensable pathway in mitotic catastrophe, is largely unknown.

The fundamental function of the centrosome, which comprises two centrioles in orthogonal configuration and surrounding pericentriolar material (PCM), is to nucleate and organize microtubules for cell migration, intracellular payload trafficking, and cell polarity in interphase and for the assembly of the mitotic spindle and the segregation of sister chromatids in mitosis [8]. The centriole is replicated once a cell cycle with template-dependent duplication, of which the cycle is remarkably similar to DNA replication, in S phase [9]. The first step of centriole duplication is the assembly of Plk4, STIL, and SAS-6 on the wall of the mother centriole [10]. Duplicated centriole pairs are separated in G2 to establish bipolar spindles by dissolving linker fibers between parental centrioles and disengaged at their proximal ends of parental centrioles by separase-mediated proteolysis of cohesin during anaphase after chromosome separation in an analogous manner. Similar to the protection of cohesin removal by Shugoshin 1 (Sgo1) and phosphatase PP2A at the centromeres in prophase, Sgo1 and a shorter Sgo1 splice variant (sSgo1) also safeguard centriole cohesion from the Plk1-mediated prophase pathway [11, 12]. In this regard, overexpression of separase or depletion of Sgo1 results in unscheduled chromosome separation and precocious centriole disengagement [13]. While supernumerary centrosomes caused by centrosome amplification (CA) through the dysregulation of the Plk4-STIL-SAS-6 module or precocious centriole disengagement are a conspicuous feature of chromosomal instability (CIN) cancers [14, 15], tumor cells undergo multipolar cell divisions by reconfiguring a multipolar spindle into a bipolar spindle through a centrosomal clustering mechanism [16, 17]. Although several DNA-damaging agents (DDAs) reportedly cause multipolar spindles [18], it is unclear how DDR triggers centrosome-mediated DiM during cell cycle progression.

During mitosis, the spindle assembly checkpoint (SAC) monitors kinetochore-microtubule attachment and spindle biorientation for faithful sister-chromatid segregation to two daughter cells [19]. In the presence of unattached or improperly attached kinetochores, the SAC delays anaphase onset via inhibition of the anaphase-promoting complex/cyclosome which activates separase through degradation of securin to cleave cohesin and, therefore, initiates sister-chromatid segregation [20]. Thus far, DDR proteins, such as ATM, ATR, Chk1, Chk2, BRCA2, MDC1, and 53BP1, have been reported to be involved in mitotic processes under unperturbed conditions [21–24] and DDR crosstalk with the SAC in DNA-damage-induced mitotic arrest [25]. For example, Chk1 phosphorylates Aurora B to delay anaphase onset in the presence of misattached kinetochores for faithful chromosome segregation by promoting BubR1 and Mps1 localization to kinetochores and to correct merotelic attachment by MCAK. Here, we report a molecular link, hornerin, that transmits signals from the DDR to the mitotic centrosome by mediating Plk1 phosphorylation by Chk1 and triggers DiM via precocious centriole disengagement and concomitant spindle multipolarity.

## Results

### DDR induces DiM via spindle multipolarity

**Fig. 1:**
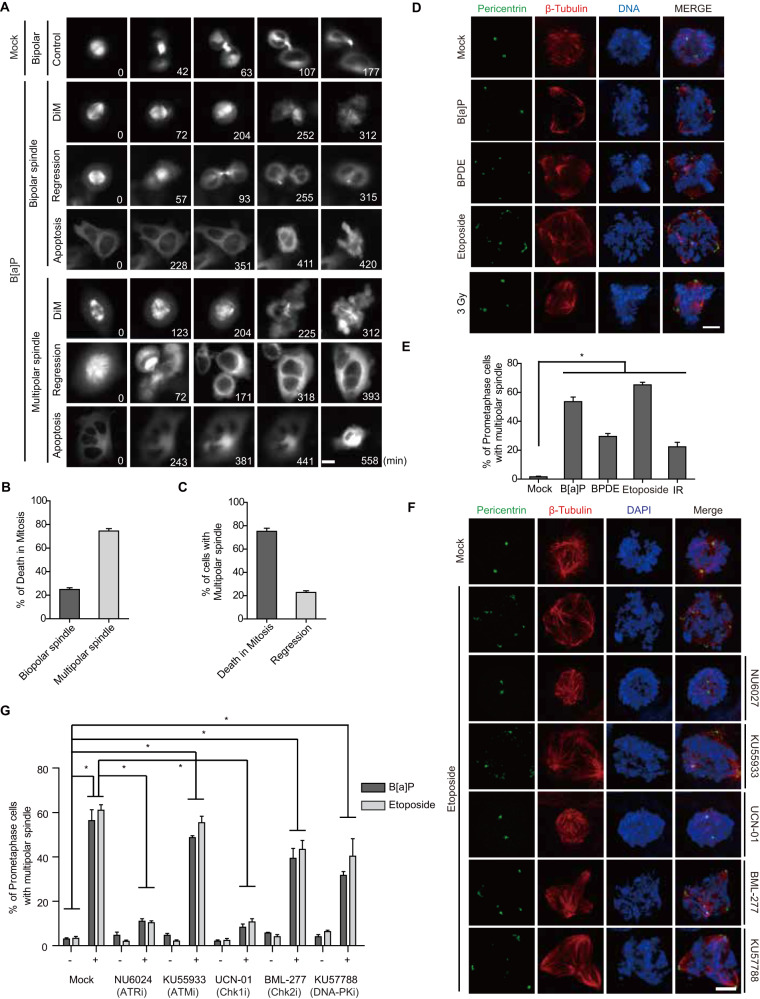
DNA damage causes multipolar spindle formation via the DDR. **A**–**C** HeLa cells treated with 0.5 µM B[a]P for 48 h were analyzed by live cell imaging for 10 h (**A**, *n* = 1609 cells). DiM (**B**, *n* = 356) and the fate of cells with multipolar spindles (**C**, *n* = 257) were analyzed. **D, E** After treatment with the indicated genotoxic stresses, HeLa cells were stained with the indicated antibodies, and the number of prometaphase cells with multipolar spindles was determined from 300 prometaphase cells in three independent experiments. **F, G** Twenty-four hours after treatment with 2 µM NU6027 as an ATR inhibitor (ATRi), 20 nM KU55933 as an ATM inhibitor (ATMi), 300 nM UCN-01 as a Chk1 inhibitor (Chk1i), 1 µM BML-277 as a Chk2 inhibitor (Chk2i), or 0.5 µM KU57788 as a DNA-PK inhibitor (DNA-PKi), HeLa cells were treated with 0.5 µM B[a]P for 48 h or 0.5 µM etoposide for 24 h, and multipolar spindles were analyzed (*n* = 400 centrioles from three independent experiments). Scale bars, 5 µm. Error bars, SEM. ^*^p < 0.01 (two-tailed *t* test).

**Fig. 2:**
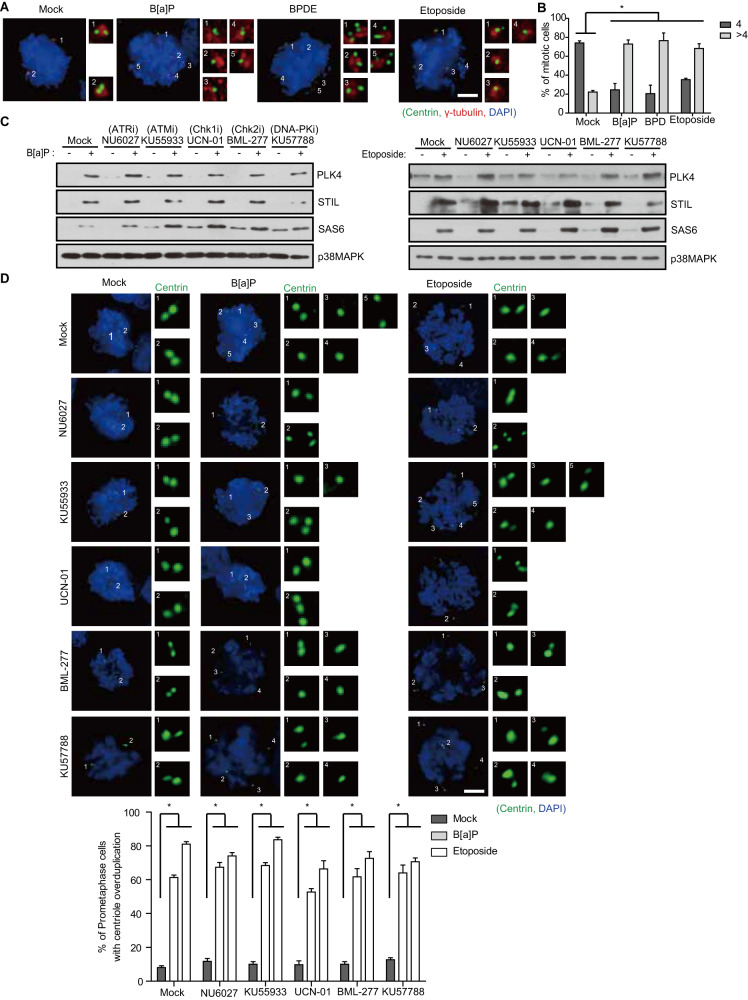
DNA damage induces centriole overduplication in a DDR-independent manner. **A, B** After treatment with the indicated chemicals, HeLa cells were stained with the indicated antibodies and the number of centrioles in prometaphase cells was analyzed and plotted (*n* = 300 cells). **C, D** Twenty-four hours after treatment with the indicated inhibitors, HeLa cells were treated with the indicated chemicals and analyzed by immunoblotting and immunostaining (**D**, *n* = 300). Images of uncropped blots are provided as a Supplementary Material file. Scale bars, 5 µm. Error bars, SEM. ^*^p < 0.01 (two-tailed *t* test).

**Fig. 3:**
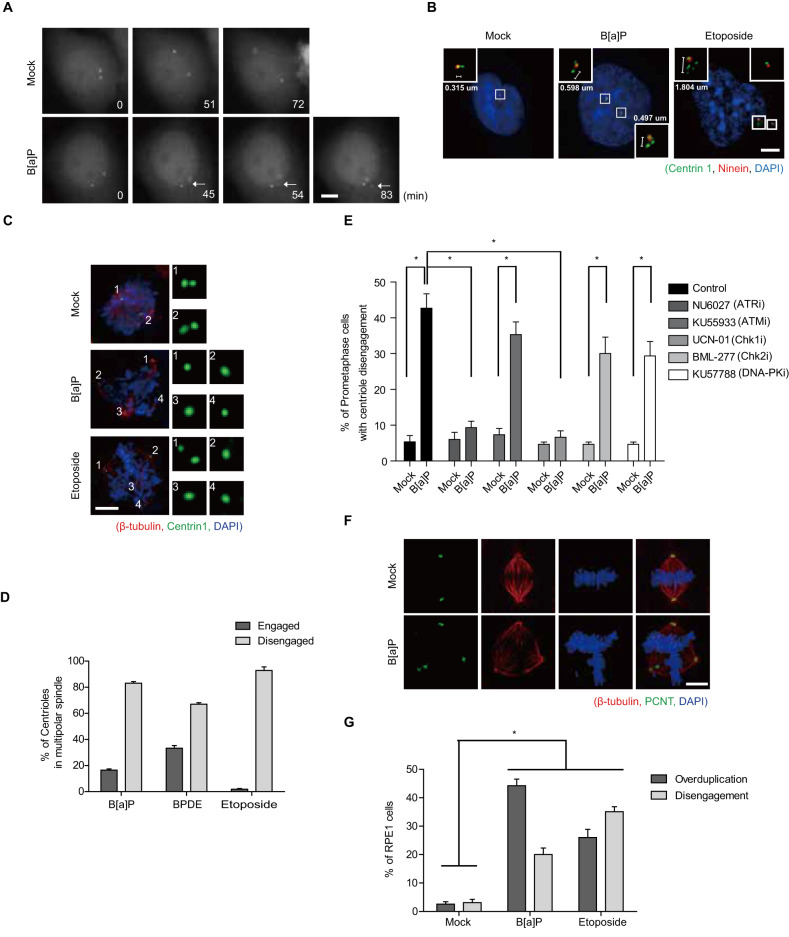
DDR induces premature centriole disengagement. **A** HeLa cells expressing GFP-centrin 2 were treated with B[a]P and analyzed by live cell imaging. Arrows point to disengaged centrioles. **B**–**E** Disengagement of centrioles in chemically treated HeLa cells was stained with the indicated antibodies and analyzed (*n* = 300). Ninein served as a marker for the mother centriole (**B**). **F, G** After treatment with the indicated DNA damaging agents, RPE-1 cells were stained with the indicated antibodies (**F**). The number of RPE-1 cells with centriole overduplication or premature centriole disengagement was analyzed as in **C** and **D** (G; *n* = 300 cells). Scale bars, 5 µm. Error bars, SEM. ^*^p < 0.01 (two-tailed *t* test).

To dissect DDR-induced mitotic catastrophe, we performed live-cell imaging with HeLa cells stably expressing GFP-tubulin after treatment with the mild DDA, benzo[a]pyrene (B[a]P), for 48 h to enrich the mitotic population and found that 70% of cells undergoing DiM harbored multipolar spindles (Fig. 1A, B, Fig. S1, and Movies 1–7). The DDA also produced polylobed progeny through regression in multipolar anaphase (Fig. 1C). DiM with a multipolar spindle configuration is thus a major pathway in mitotic catastrophe because most genotoxic agents drive the formation of multipolar mitotic spindles through the ATR-Chk1 pathway in the DDR (Fig. 1D, E, F, G), as previously described [26, 27]. Because B[a]P is known as a ligand for the aryl hydrocarbon receptor (AhR) and aggravates its own toxicity as a DDA by enhancing metabolic enzymes including CYP1A1, 1A2, and 1B1 [28], depletion of AhR expectedly decreased phospho-H2AX positive mitotic cells under B[a]P treatment and concurrently rescued B[a]P-induced multipolarity (Fig. S2). In contrast, etoposide-induced DNA damage and concomitant multipolarity were not rescued by AhR depletion, indicating that AhR is not directly involved in DDR-induced multipolarity. Although several abnormal conditions, such as centriole overduplication, precocious centriole disengagement, and pericentriolar material (PCM) fragmentation, clearly result in CA and the formation of multipolar spindles [18, 29], the mechanism underlying DDR-induced spindle multipolarity is not yet clear. Consistent with previous reports [30], DDAs caused centriole overduplication in G2 and ultimately generated CA during mitosis (Fig. 2A, B). Given that Plk4, STIL, and SAS-6 are critical for the initiation of centriole duplication [10], we examined protein levels after treatment with DDAs and found substantial increases in these three proteins (Fig. 2C). In stark contrast to spindle multipolarity, the increases in Plk4, STIL, and SAS-6 and DNA damage-induced centriole overduplication were not suppressed by any of the inhibitors of the DDR (Fig. 2C, D), indicating that the DDR induces centriole overduplication in an ATM/ATR-independent manner. Because the radiomimetic drug, doxorubicin, and replication stress reportedly cause precocious centriole disengagement in G2 and mitosis [31–33], we examined centriole configuration by time-lapse imaging of HeLa cells stably expressing GFP-centrin after treatment with DDAs. As expected, B[a]P caused premature centriole disengagement in G2, which in turn resulted in the formation of multipolar spindles during mitosis (Fig. 3A, B, C, D, and Movies 8, 9). Intriguingly, inhibition of ATR or Chk1 substantially rescued DNA damage-induced premature centriole disengagement (Fig. 3E), strongly suggesting that the DDR triggers multipolar spindle and concomitant DiM via precocious centriole disengagement rather than centriole overduplication. Similar perturbation of centrosome and centriole integrity by DDAs was observed in untransformed RPE-1 cells (Fig. 3F, G, and Fig. S3).

### DDR interferes with the targeting of Plk1 and Sgo1 to the centrosome

**Fig. 4:**
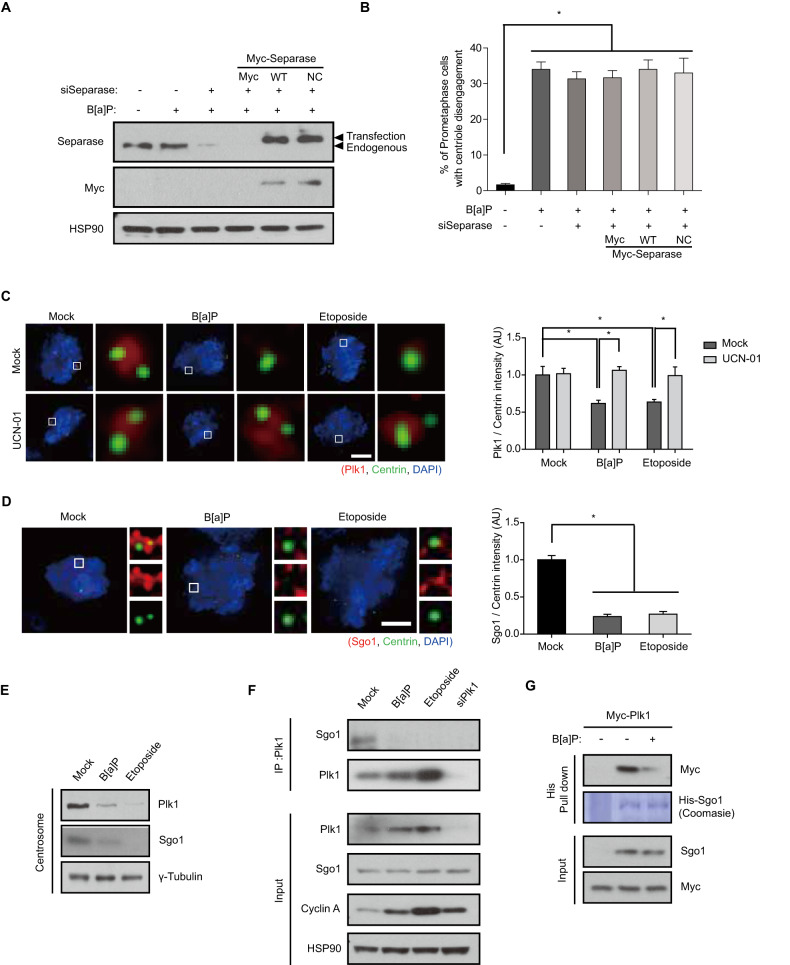
DDR disrupts the translocation of Plk1 and Sgo1 to the centrosome. **A, B** Twenty-four hours after siRNA transfection, HeLa cells were transfected with plasmids containing WT or noncleavable (NC) mutant separase and treated with B[a]P for 48 h. The lysates were analyzed by Western blotting with the indicated antibodies. Prometaphase cells with centriole disengagement were counted, and the data were plotted (*n* = 300). **C, D** The intensity of Plk1 and Sgo1 in the centrosome after genotoxic stress treatment (*n* = 30). **E** Forty-eight hours after treatment with the indicated chemicals, centrosomes were purified and analyzed by Western blotting with the indicated antibodies. **F** Immunoprecipitates from chemically treated HeLa cells were analyzed by immunoblotting. **G** Lysates from plasmid transfected cells were incubated with Ni-beads immobilized with His-Sgo1. Pulled-down proteins were analyzed by immunoblotting. Images of uncropped blots and gels are provided as a Supplementary Material file. AU, arbitrary units, Scale bars, 5 µm. Error bars, SEM. ^*^p < 0.01 (two-tailed *t* test).

We speculated that separase and Plk1 could be relevant targets of the DDR in DiM because both are best known for their roles in relieving sister chromatid and centriolar cohesion during the metaphase-anaphase transition and are associated with precocious centriole disengagement in perturbed conditions [11, 34]. DNA damage-induced precocious centriole disengagement was not reversed by the depletion of separase with siRNA targeting the 3’ noncoding sequence of the endogenous separase gene (Fig. 4A, B). However, in addition to inhibition of Plk1 activity [35], immunofluorescence microscopy showed that the localization of Plk1 in the centrosome substantially decreased in a Chk1-dependent manner after treatment with DDAs (Fig. 4C). Given that Plk1 phosphorylates not only the SA/Scc3 cohesin subunit to trigger cohesin dissociation from the chromosome arm and centriole without proteolytic activity of separase in the prophase pathway but also Sgo1 to target it to the centrosome and protect centriole cohesion during early mitosis [12, 36–39], we sought to investigate the relationship between Plk1 and Sgo1 in DDR-induced precocious centriole disengagement. Interestingly, the translocation of Sgo1 to the centrosome was drastically diminished in cells treated with the DDAs (Fig. 4D, E) and the interaction of Plk1 with Sgo1 was substantially decreased in an immunoprecipitation assay and an in vitro binding assay with cell lysates from cells treated with the DDAs (Fig. 4F, G). These data indicate that the DDR disrupts the interaction of Plk1 with Sgo1 to induce precocious centriole disengagement.

### Chk1 phosphorylates the polo-box domain of Plk1

**Fig. 5:**
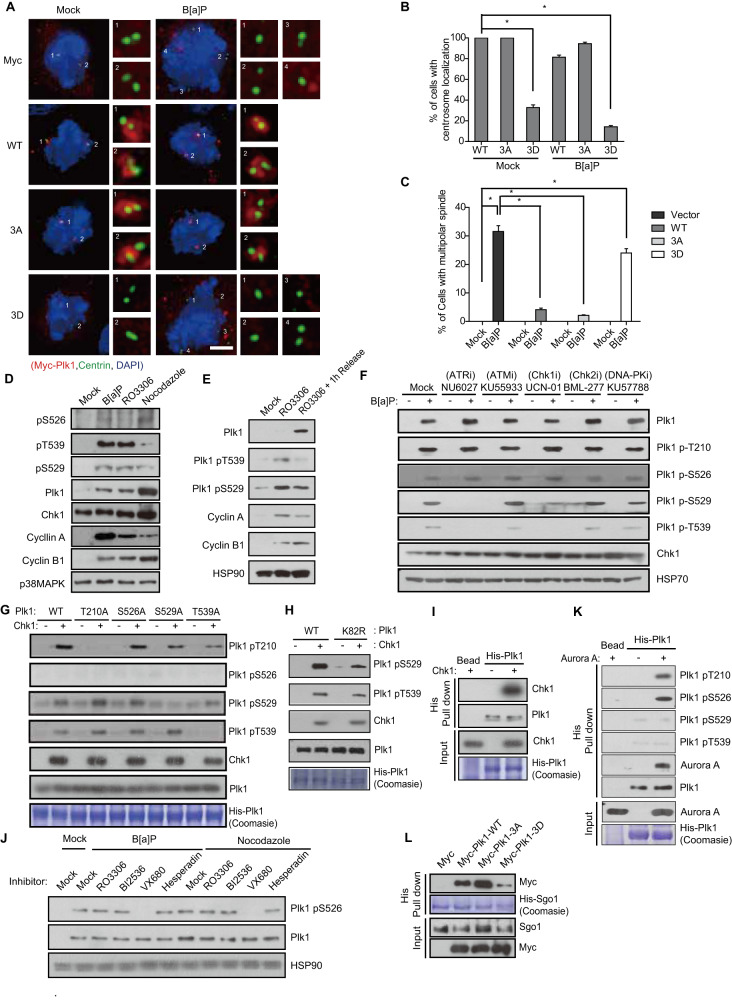
Chk1 directly phosphorylates Plk1 to disturb its interaction with Sgo1. **A**–**C** After transfection of plasmids and B[a]P treatment, the localization of myc-Plk1 and multipolar spindles was analyzed (*n* = 300). **D, E** Chemically treated HeLa cells were analyzed by immunoblotting. **F** Cells were treated with the indicated inhibitors and B[a]P and analyzed by immunoblotting. **G, H** WT or mutant His-Plk1 was incubated with active Chk1 and analyzed by immunoblotting. **I** Recombinant Chk1 was incubated with recombinant His-Plk1 and proteins pulled down with Ni-beads were analyzed by immunoblotting. **J** After treatment with 0.5 µM B[a]P for 48 h or 300 ng/ml nocodazole for 16 h, cells were treated with 1 µM RO3306 as a Cdk1 inhibitor for 5 h, 1 µM BI2536 as a Plk1 inhibitor for 1 h, 5 µM VX680 as an Aurora A inhibitor for 1 h, and 2 µM Hesperadin as an Aurora B inhibitor for 5 h. Cell lysates were analyzed by immunoblotting. **K** His-Plk1 was incubated with Aurora A, pulled down with Ni^+^-beads, and analyzed by immunoblotting. **L** Lysates from DNA-transfected HeLa cells were incubated with recombinant His-Sgo1 and proteins pulled down with Ni-beads were analyzed by immunoblotting. Images of uncropped blots and gels are provided as a Supplementary Material file. Scale bar, 5 µm. Error bars, SEM. ^*^p < 0.01 (two-tailed *t* test).

To investigate the mechanism underlying the disruption of Plk1 translocation to the centrosome by Chk1, we sought to identify phosphorylation sites within the polo-box domain (PBD), which is responsible for both the substrate specificity and centrosomal localization of Plk1 [40–42], by immunoprecipitation with mass spectrometry (Fig. S4A). We identified Ser^526^, Ser^529^, and Thr^539^ in the PBD as candidate sites among 14 phosphorylation residues under B[a]P-treatment (Fig. S4B). As expected, changing three residues to phosphorylation-mimicking Asp (3D) abrogated the localization of Plk1 to the centrosome, whereas changing these residues to Ala (3A) did not affect targeting to the centrosome (Fig. 5A, B). We consistently found that the WT and 3 A mutant, but not the 3D mutant, successfully rescued spindle multipolarity under B[a]P-treatment (Fig. 5C). Next, we generated phospho-Ser^526^-specific, phospho-Ser^529^-specific, or phospho-Thr^539^-specific antibodies to confirm the phosphorylation of endogenous Plk1 and determine the corresponding kinase (Fig. S4C). Whereas three residues were phosphorylated in B[a]P-treated cells, Ser^529^ and Thr^539^ were phosphorylated mainly in Cdk1 inhibitor-treated cells arrested in the G2 phase and Ser^526^ in nocodazole-treated cells arrested in early mitosis (Fig. 5D). Consistent with this, phosphorylation of Ser^529^ and Thr^539^ decreased in cells released from G2 arrest (Fig. 5E). These data indicate that individual phosphorylation of Ser^526^, Ser^529^ and Thr^539^ is involved in cell cycle progression during G2 and mitosis. Similar to the 3D mutant, phosphorylated Plk1 also decreased at the centrosomes after treatment with DDA (Fig. S4D). Because the level of Plk1 at the centrosome was recovered by the Chk1 inhibitor (Fig. 4C), we tested whether Chk1 was the corresponding kinase for phosphorylation of the PBD in Plk1. Consistent with our findings, both ATR and Chk1 inhibitors abolished the phosphorylation of Ser^529^ and Thr^539^ induced by B[a]P treatment (Fig. 5F). In addition to phosphorylation of Thr^210^ during mitosis [43], Chk1 directly phosphorylated Ser^529^ and Thr^539^ in an in vitro kinase assay (Fig. 5G). To exclude the possibility of autophosphorylation by active Plk1, we confirmed the phosphorylation of a dominant-negative lysine 82 to arginine mutated Plk1 (K82R) [44] by Chk1 (Fig. 5H). Furthermore, mutation of Ser^529^ and Thr^539^ to Ala abrogated specific phosphorylation by Chk1 (Fig. 5G) and Chk1 directly interacted with Plk1 in an in vitro binding assay (Fig. 5I). These data indicate that Chk1 is a physiological kinase for phosphorylation at Ser^529^ and Thr^539^ in the PBD of Plk1 under DDR. However, the phosphorylation of Ser^526^ was not abolished by either ATR or Chk1 inhibitors and was not induced by Chk1 (Fig. 5F, G). Because the phosphorylation of Ser^526^ was substantially increased in nocodazole-treated cells arrested in early mitosis (Fig. 5D), we investigated whether mitotic kinases participate in this phosphorylation. Intriguingly, the Aurora A inhibitor, but not inhibitors against mitotic kinases including Cdk1, Plk1, and Aurora B, drastically abolished the phosphorylation of Ser^526^ (Fig. 5J). In addition to phosphorylation at Thr^210^ in late G2 and early mitosis [45], Aurora A strongly phosphorylated Ser^526^ in an in vitro kinase assay (Fig. 5K), suggesting that Aurora A is involved not only in Plk1 activation via Thr^210^ phosphorylation upon mitotic entry but also in mitotic catastrophe via Ser^526^ phosphorylation in mitotic cells harboring DNA damage. Strikingly, the interaction of the 3D mutant with Sgo1 substantially decreased in an in vitro pull-down assay (Fig. 5L), strongly suggesting that DDR-mediated phosphorylation of the PBD by Chk1 disrupts the interaction with Sgo1 and its translocation to the centrosome.

### Hornerin forms a complex with Plk1 and Chk1 to mediate Plk1 phosphorylation

**Fig. 6:**
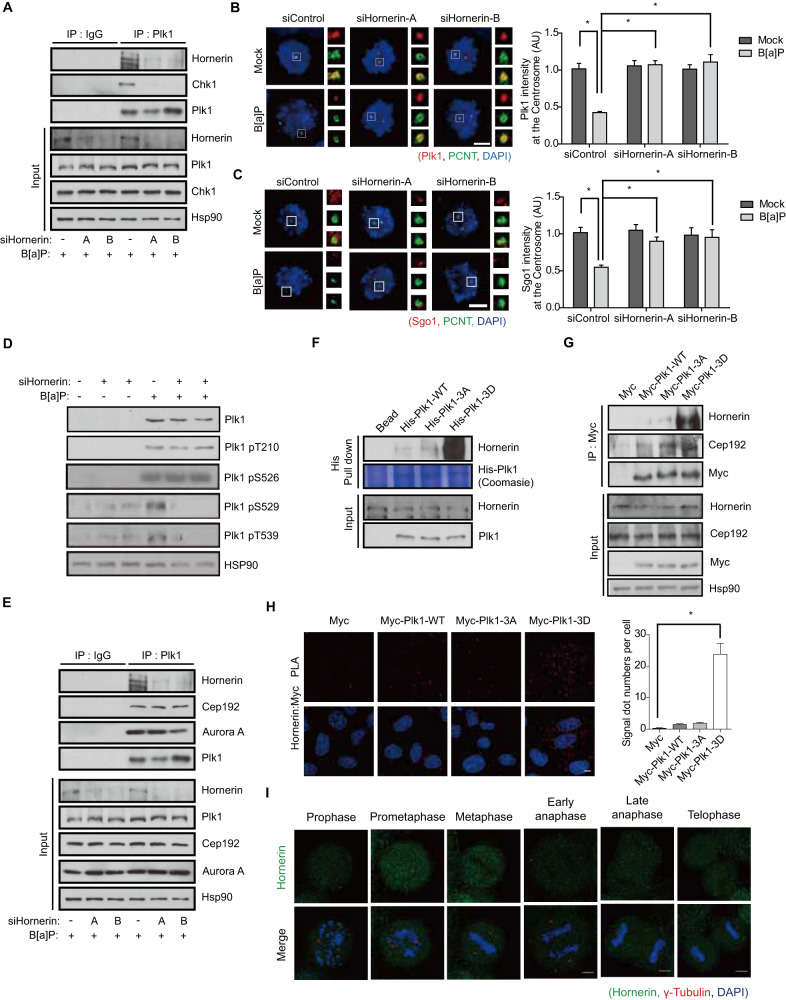
Hornerin mediates Plk1 phosphorylation by Chk1. **A–D** siRNA-transfected HeLa cells were treated with B[a]P and analyzed by IP-immunoblotting (A and E), immunostaining (B and C; *n* = 300), and immunoblotting (D). **E** siRNA-transfected HeLa cells were treated with B[a]P and analyzed by IP-immunoblotting. **F** Twenty-four hours after transfection of WT or mutant Plk1, the cell lysates were incubated with recombinant His-Plk1 and proteins pulled down with Ni-beads were analyzed by immunoblotting. **G** HeLa cells transfected with the indicated plasmid were subjected to IP-immunoblotting. **H** Hornerin-Plk1 3D proximity by in situ PLA in HeLa cells transfected with myc-Plk1 WT or mutants (*n* = 30). **I** HeLa cells were stained with hornerin (green), γ-tubulin (red), and DAPI (blue). Images of uncropped blots and gels are provided as a Supplementary Material file. AU, arbitrary units, Scale bars, 5 µm. Error bars, SEM. ^*^p < 0.01 (two-tailed *t* test).

To delineate the mechanism underlying DDR-induced DiM, we next sought to identify a mediator between Chk1 and Plk1 through purification of the Plk1 complex and found hornerin via mass spectrometry (Fig. S5A, B). Analysis of a deletion mutant of hornerin revealed that Chk1 and Plk1 bound to the N-terminus and C-terminus of hornerin, respectively, to form an integrative complex between the DDR and DiM (Fig. S5C, D, E, F). Consistent with this idea, the interaction between Chk1 and Plk1 was abolished by hornerin-depletion (Fig. 6A). Furthermore, the levels of Plk1 and Sgo1 at the centrosome were recovered by the depletion of hornerin in B[a]P treated cells (Fig. 6B, C), presumably because hornerin mediates the phosphorylation of Plk1 by Chk1. Indeed, the phosphorylation of Plk1 at Ser^529^ and Thr^539^, but not Ser^526^, after B[a]P treatment was dramatically diminished in hornerin-depleted cells (Fig. 6D), suggesting that Ser^529^ and Thr^539^ are phosphorylated by Chk1 in a DDR- and hornerin-dependent manner but that Ser^526^ is phosphorylated by Aurora A in a DDR- and hornerin-independent manner. Consistently, overexpression of WT or siRNA-resistant hornerin (Myc-Hornerin-R) restored the ability of B[a]P-treated cells to form multipolar spindles in hornerin-depleted cells, as both siHornerin-A and siHornerin-B target the noncoding sequence of the endogenous hornerin gene (Fig. S6A, B, C, D, E). However, depletion of hornerin did not dissociate the Cep192::Aurora A::Plk1 complex (Fig. 6E). Therefore, DNA damage response did not interfere with the translocation of Cep192-AurA-Plk1 complex to the centrosome and could not induce the centriole disengagement in hornerin-depleted cells (Fig. 6B). Because DNA damage response could not eliminate mitotic cells with DNA damages through DiM without hornerin, depletion of hornerin increased the number of metaphase cells harboring damaged DNA by incapacitating DDR-induced DiM (Fig. S7). Intriguingly, the Plk1 3D mutant strongly interacted with hornerin in an in vitro pull-down assay, an immunoprecipitation assay, and proximity ligation assays (in situ PLAs) (Fig. 6F, G, H), possibly explaining the impaired translocation of Plk1 to the centrosome through trapping phosphorylated Plk1 by hornerin in the cytoplasm under B[a]P treatment. As expected, hornerin did not localize at the centrosome but exhibited diffusive localization in the cytoplasm (Fig. 6I). Hornerin-mediated multipolarity is not a cell type- or tissue-specific mechanism because similar centrosome perturbation by DDAs was observed in osteosarcoma epithelial cells, such as U2OS cells (Fig. S8A). Although skin cancer cells exhibited resistance against B[a]P probably because of their capacity of DNA repair, we also confirmed hornerin-mediated multipolarity in melanomas, including SK-MEL-2 and SK-MEL-28 cells, with etoposide (Fig. S8B, C).

### DDR inactivates Plk1 through Chk1-mediated phosphorylation of the PBD

**Fig. 7:**
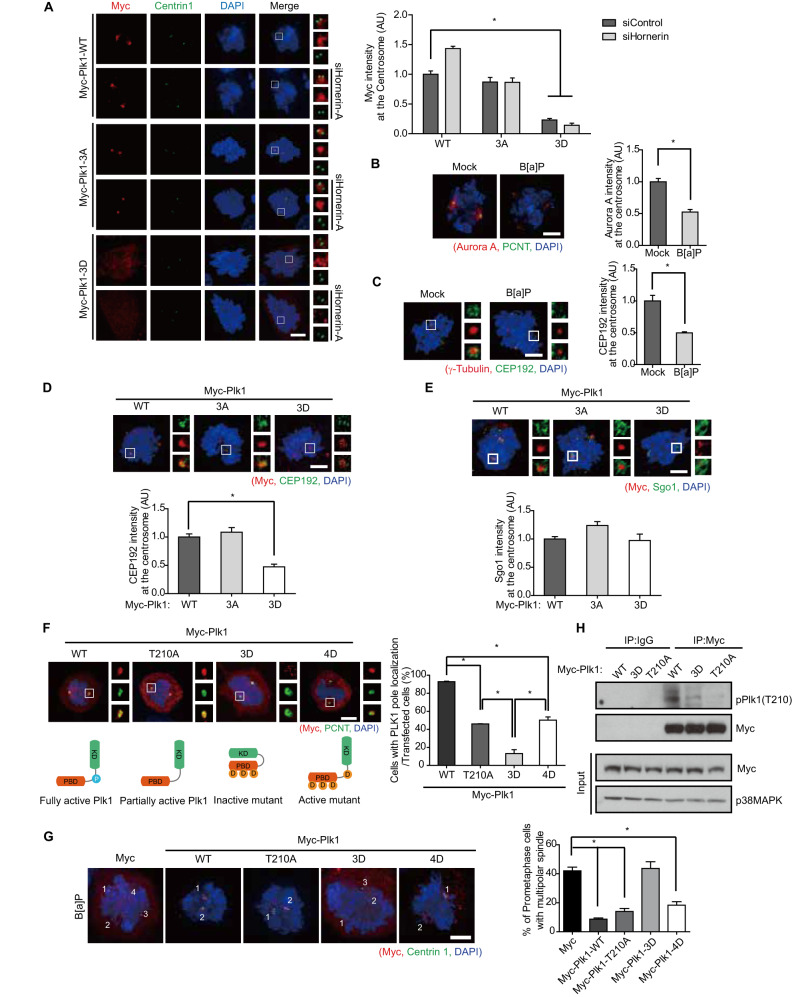
DDR abrogates Plk1 activation and translocation. **A** Twenty-four hours after siRNA-transfection, HeLa cells were transfected with the indicated plasmids and analyzed by immunostaining (*n* = 300). **B, C** HeLa cells were treated with B[a]P and analyzed by immunostaining (*n* = 300). **D**–**G** HeLa cells transfected with the indicated plasmid were analyzed by immunostaining (*n* = 300). **H** Twenty-eight hours after transfection of WT or mutant Plk1, the cells were treated with B[a]P for two hours and then analyzed by immunoblotting. Images of uncropped blots are provided as a Supplementary Material file. KD, kinase domain. AU, arbitrary units, Scale bar, 5 µm. Error bars, SEM. ^*^p < 0.01 (two-tailed *t* test).

Whereas the level of WT Plk1 was increased at the centrosome by neutralizing the sequestration of phosphorylated Plk1 through hornerin depletion, the level of the 3D mutant in the centrosome was not recovered in hornerin-depleted cells (Fig. 7A). Given that Cep192 recruits Plk1 and Aurora A to the centrosome [46] and that the kinase activity of Plk1 is pivotal for Cep192 targeting to the centrosome [47], we reasoned that the phosphorylation of the PBD by Chk1 might affect Plk1 activation. In agreement with our hypothesis, the levels of Cep192 and Aurora A in the centrosome were decreased in B[a]P treated cells (Fig. 7B, C). Furthermore, because the 3D mutant, similar to the WT and 3 A mutant, interacted with Cep192 (Fig. 6G), the level of Cep192 was decreased in the centrosome via sequestration with hornerin due to the dominant negative effect of the 3D mutant (Fig. 7D). In contrast, overexpression of the 3D mutant did not decrease the level of Sgo1, as endogenous Plk1 targets Sgo1 to the centrosome and, therefore, did not cause multipolar spindles (Figs. 7E, 5C). As both PBD docking-induced conformational changes and phosphorylation of Thr^210^ in the T-loop activate Plk1 [48], we examined the centrosome localization of T210A and 4D (T210D, S526D, S529D, and T539D) mutants to investigate the reason for the impaired targeting of the Plk1 3D mutant even in the absence of hornerin. Interestingly, both the T210A and 4D mutants exhibited half centrosome targeting and rescue capacity similar to that of WT (Fig. 7F, G). Furthermore, Thr^210^ in the 3D mutant was not phosphorylated (Fig. 7H), indicating that the phosphorylation of the PBD by the DDR completely disrupts the activation of Plk1 through conformational change and disturbing Thr^210^ phosphorylation. Indeed, centrosome separation in G2 was perturbed in DDA-treated cells (Fig. S9), which is reminiscent of the depletion of Plk1 protein or the inhibition of Plk1 activity. The delay in mitotic progression caused by perturbation of centrosome separation presumably induces DiM in cells harboring DNA damage and bipolar spindles (Fig. 1B). Because Ser^137^ and Trp^414^ are reportedly essential for Thr^210^ phosphorylation and centrosome targeting of Plk1 [49–51], we examined the interplay among these residues. While S137A or S137D mutants localized to the centrosome with perturbed centrosome integrity, the addition of a S137A mutation into the 4D mutant disrupted localization to the centrosome (Fig. S10A), suggesting that the phosphorylation of Ser^137^ is essential for the centrosome localization of Plk1 and the centrosome integrity. Next, we investigated whether the addition of S137D mutation into the 3D mutant rescues the centrosome localization of 3D mutant by inducing Thr^210^ phosphorylation. Unexpectedly, the S137D mutation in 3D mutant did not recover the Thr^210^ phosphorylation suppressed by 3D mutation and the centrosome localization of 3D mutant (Fig. S10A, B). These data indicate that phosphorylation of Ser^137^ is also required for the centrosomal function of Plk1 and that triple phosphorylation of PBD invalidates the function of phosphorylation at Ser^137^ for centrosome targeting of Plk1. Consistent with a previous report, the addition of the W414F mutation into the 4D mutant disrupted centrosome targeting of Plk1, suggesting that Trp^414^ is essential for centrosome targeting of Plk1 (Fig. S10A). Together, our results provide strong evidence that DDR-hornerin-mediated Plk1 phosphorylation at the PBD disturbs Plk1 activation and concurrent translocation of the Cep192::Aurora A::Plk1 complex and Sgo1 to the centrosome to induce spindle multipolarity and concomitant mitotic catastrophe.

### Hornerin governs DDR-induced DiM

**Fig. 8:**
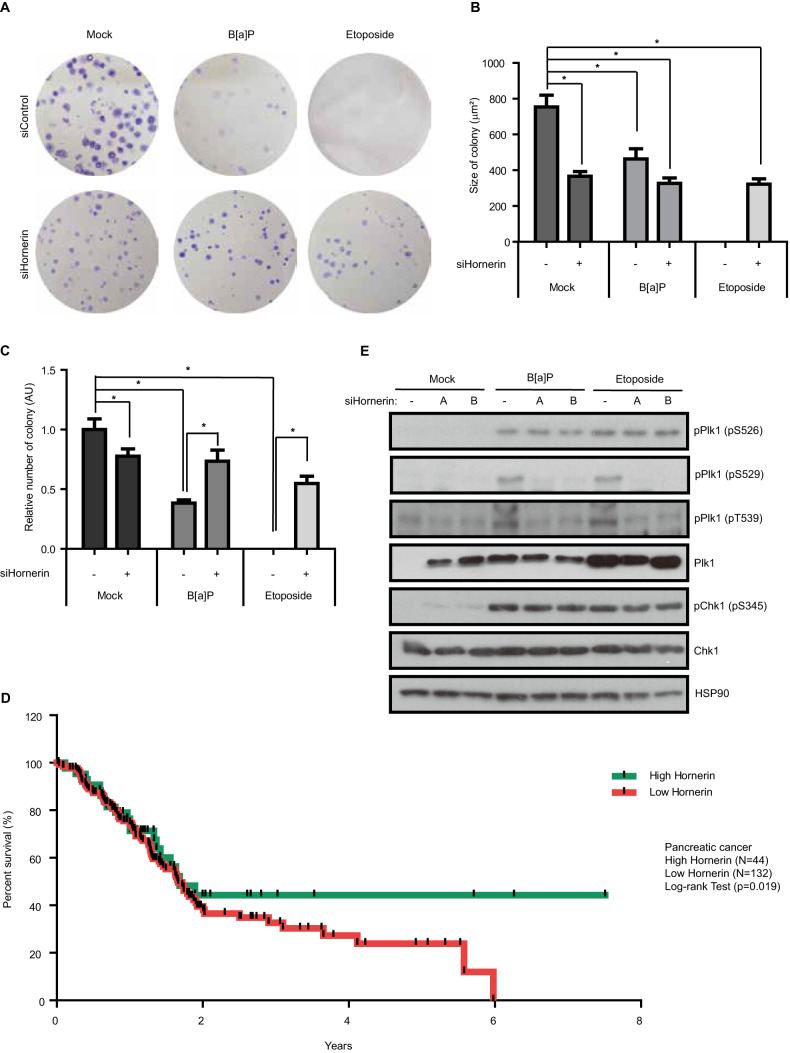
Hornerin is indispensable for DDR-induced mitotic catastrophe. **A** Colony formation assay (14 days) following hornerin knockdown. The image is representative of three independent experiments. **B** The formation of colonies by B[a]P (0.5 µM, replaced every three days) or etoposide (1 µM, replaced every three days) was quantified and plotted. **C** The size of colonies was quantified and plotted (*n* = 100 colonies from three independent experiments). **D** The survival curves of patients stratified according to the expression of hornerin mRNA in the TCGA database. Log-rank P value for Kaplan-Meier plot showing results from analysis of correlation between mRNA expression level and patient survival. **E** HeLa cells were treated with the indicated DDAs and analyzed by immunoblotting. AU, arbitrary units, Error bars, SEM. ^*^p < 0.01 (two-tailed *t* test).

We have described a molecular pathway leading to DiM via DDR. Our data also show a dissection between DiM with multipolar spindles and binucleation in mitotic catastrophe. Interestingly, the size of the colonies in the clonogenic assay was decreased by depletion of hornerin (Fig. 8A, B). Because individual phosphorylation at Ser^526^, Ser^529^ or Thr^539^ in PBD of Plk1 was detected in G2 or mitosis under unperturbed conditions (Fig. 5D, E), we propose that single or double phosphorylation in PBD is required for cell cycle progression. As DDR typically induces different types of cell death, including apoptosis, necroptosis, and mitotic catastrophe, in compliance with the type of damage and cell cycle stage [52, 53], we speculated that the level of hornerin-mediated DiM might not exceed the level of cell death in interphase. To directly test this hypothesis, we evaluated the proportion of cells that underwent hornerin-mediated DiM among all cells that underwent DDA-induced cell death. Strikingly, however, depletion of hornerin drastically increased the cell survival rate by over 50% compared to that of control cells treated with etoposide in long-term clonogenic assays (Fig. 8A, C). Consistent with this, the mRNA expression level of hornerin showed the strongest correlation with the survival rate of patients with cervical, pancreatic, or renal cancer (Fig. 8D and Fig. S11). Because depletion of hornerin specifically abrogated PBD phosphorylation in Plk1 but not active phosphorylation of Chk1 (Figs. 6D, 8E), we conclude that hornerin-mediated DiM is one of the major-types of DDR-induced cell death for eliminating damaged cells throughout the cell cycle.

## Discussion

Here, we identify the molecular mechanism by which hornerin, which is a component of the epidermal differentiation complex and contributes to envelope cornification and the epidermal antimicrobial barrier [54, 55], mediates the phosphorylation of the PBD in Plk1 by Chk1 to link DDR to DiM through premature centriole disengagement. We demonstrate that hornerin controls Plk1 and Sgo1 recruitment to centrosomes upon DNA damage by regulating Chk1-mediated phosphorylation of Plk1 (Fig. 6). Importantly, our mutational studies uncovered three critical residues in Plk1 required for the sequestration of Plk1 by hornerin, the intervention of Plk1 interaction with Sgo1, and the suppression of Plk1 activation by inhibiting T-loop phosphorylation and conformational change (Fig. 7F, G). Depletion of Plk1 and Sgo1 in centrosomes caused by Chk1-mediated Plk1 phosphorylation results in premature centriole disengagement in G2 and concurrent multipolar spindles in mitosis, leading to DiM.

Maintenance of genome stability, which is safeguarded by the precise orchestration of DNA replication, DNA repair, chromosome segregation, and cell cycle checkpoints, is the primary objective of cell cycle progression. Because DNA is replicated, compacted, and untangled through the cell cycle, chromatin, the substrate of DNA repair, undergoes structural transitions along with cell cycle progression. In this regard, the cell cycle restricts the type of DNA repair in distinct cell cycle phases. While nonhomologous end-joining (NHEJ) is the major repair pathway for DNA double strand breaks (DSBs) in G1, homologous recombination (HR) is restricted to the S and G2 phases because the ideal template of HR is the sister chromatid [56, 57]. Although mitotic cells permit minimal DNA repair for Holliday junction (HJ) dissolution and resolution [58], the major DNA repair pathways, including NHEJ and HR, are suppressed by Cdk1-mediated phosphorylation of the E3 ubiquitin ligases RNF8 and 53BP1 to prevent sister telomere fusions during mitosis [3]. However, mitotic cells sense all types of DSBs and initiate a limited DDR signaling cascade to mark their DNA damage for repair after mitotic exit [59]. Whereas NBS1 and Ku localize to mitotic DSBs and H2AX is phosphorylated in an ATM- and DNA-PK-dependent manner, the signaling cascade downstream of MDC1, including RNF8, RNF168, BRCA1, and 53BP1, fails to colocalize with γH2AX [60, 61]. In addition, Plk1 inhibits the DDR in mitosis by phosphorylating Chk2 and mediating the degradation of claspin, the adaptor protein of Chk1, through β-TrCP-SCF [62, 63]. Conversely, Chk1 phosphorylates Thr^210^ to activate Plk1 in unperturbed mitosis [43]. As an abrogated or compromised G2 checkpoint allows premature mitotic entry of defective cells harboring DNA damage or incompletely replicated DNA thereby leading to mitotic catastrophe [64–66], the crucial question arises as to how DDR induces DiM after entry into mitosis. DNA damage progressing from failed earlier checkpoints could provoke destructive cellular architecture during mitosis [26, 67, 68]. Our results, along with previous observations, indicate that premitotic DNA damage triggers DiM through ATR-Chk1-dependent premature centriole disengagement in G2 and concomitant multipolar spindle formation in mitosis (Fig. 3). Despite various inhibition of DNA repair and DDR, DNA damage induced in mitosis affects mitotic progression as severe DNA damage appears to induce a SAC-dependent but DDR-independent mitotic delay and concomitant mitotic catastrophe [69–71].

In addition to its primary function as a center of microtubule nucleation in both interphase and mitosis, centrosomes communicate with the DDR apparatus during cell cycle progression [18]. The initial activation of Cdk1, which occurs in the centrosome prior to the activation of nuclear Cdk1 and concomitant mitotic entry, is inhibited by Chk1- or Chk2-mediated phosphorylation of centrosomal CDC25 phosphatase upon DNA damage [72–74]. Furthermore, a number of DDR proteins, such as ATM, ATR, Chk1, Chk2, BRCA1, BRCA2, and poly(ADP-ribose) polymerases (PARPs), have been found at centrosomes during distinct phases of the cell cycle [18]. Thus, the centrosome is the center of cell fate determination throughout the cell cycle, as interdependent pathways of the DDR and cell cycle intersect at centrosomes. Our observations show that hornerin-mediated DiM, of which the mechanism is Plk1 phosphorylation by Chk1 and Aurora A and concurrent structural disintegration of the spindle, is the major pathway to prevent inheritance of damaged DNA in mitotic exit (Figs. 1B, 8C). In this context, the gain of centrosome clustering function endows cancer cells with the ability to escape DiM because supernumerary centrosomes are the most common feature in many tumor tissues, high-grade and recurrent tumors, and cancer cell lines [75–77]. Moreover, perturbed expression of hornerin possibly provides a starting point for the emergence of DDA-resistant cancer cells due to cancer-specific DDR defects, as depletion of hornerin substantially increased the survival rate of etoposide-treated cells (Fig. 8A, C). Thus, hornerin-mediated DiM, as the checkpoint in mitotic exit, is clinically relevant and could be exploited to improve therapeutic outcomes for cancer patients (Fig. 8D and Fig. S11).

**Fig. 9:**
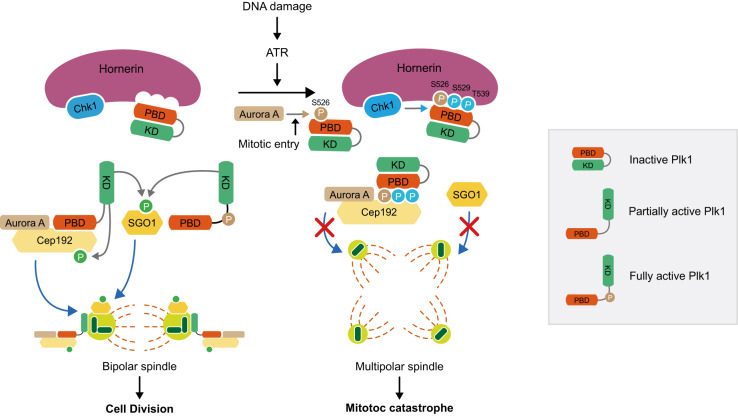
Model of hornerin-mediated mitotic catastrophe. Upon mitotic entry with DNA damage, Aurora A phosphorylates Ser^526^ for normal mitotic progression but hornerin mediates additional phosphorylations by Chk1 at Ser^529^ and The^539^ in Plk1 to induce multipolar spindle and concomitant mitotic catastrophe. KD kinase domain.

We established bifurcated pathways to induce mitotic catastrophe. The primary pathway is represented by hornerin which links the DDR to DiM by mediating the phosphorylation of Plk1 by Chk1 and the concurrent depletion of Sgo1 and the Cep192::Aurora A::Plk1 complex at the centrosome (Fig. 9). Cells with DNA damage but evading DiM are eradicated by Aurora B-mediated activation of the abscission checkpoint [7]. DDR-induced DiM, as the safeguard at the end of the cell cycle, ensures the faithful inheritance of genetic information to the next generation of cells.

## Materials and methods

### Plasmids

GFP-tagged Sgo1 expression vector, myc-tagged Separase expression vector, and pCer-C3-Plk1-K82R vector were purchased from Addgene (Watertown, MA). Point mutants of Plk1 (S526D, S529D, T539D, S526D/S529D, S526D/T539D, S529D/T539D, S526D/S529D/T539D, T210DS526D/S529D/S539D, T210A, S529A, T539A, or S526A/S529A/T539A) were generated using a Muta-directTM site-directed mutagenesis kit (iNtRON BIOTECHNOLOGY, Seongnam, Korea) according to the manufacturer’s protocol. To generate the His-Plk1 or His-Sgo1 vector, the PCR product of the human Plk1 or Sgo1 gene was cloned into the PET-28a vector (Novagen, Darmstadt, Germany).

### Antibodies

Rabbit antibodies against phosphorylated Ser^529^ and phosphorylated Thr^539^ in Plk1 were generated using C-AIILHLSNG(p)SVQIN and QINFFQDH(p)TKLILC phosphor-peptides, respectively (AbClon, Seoul, Korea). Rabbit antibodies against phosphorylated Ser^526^ were generated using L(p)SNGSVQI-C phospho-peptide (GenScript, New Jersey, USA). For Western blotting, antibodies against the following were used (clone name, dilution, manufacturer and catalog number in parentheses): cyclin A (B-8, 1:1000, Santa Cruz Biotechnology Inc, sc-271682), cyclin B1 (H-20, 1:1000, Santa Cruz Biotechnology Inc., sc-594), p38 MAPK (N-20, 1:1000, Santa Cruz Biotechnology Inc., sc-728), Sgo1 (1:1000, Thermo Scientific Pierce Antibodies, PA5-30869), β-tubulin E7 monoclonal antibody (1:1000, Developmental Studies Hybridoma Bank USA, E7), separase (XJ11-1812, 1:500, Abcam, ab16170), PLK4 (6H5, 1:500, Millipore, MABC544), SAS-6 (91.390.21, 1:100, Santa Cruz Biotechnology Inc., sc-81431), STIL (1:500, Abcam, ab89314), Chk1 (2G1D5,1:2500, Cell Signaling Technology, #2360), c-Myc (9E10, 1:1000, Santa Cruz Biotechnology Inc., sc-40), HSP70 (3A3, 1:10000, Santa Cruz Biotechnology Inc., sc-32239), hornerin (1:500, NOVUS Biologicals, NBP1-80807), AhR (1:1000, Proteintech, 67785-1), purified Plk1 pT539 (1:500, AbClon), and purified Plk1 pS529 (1:500, AbClon). For immunostaining, antibodies against the following were used (clone name, dilution, manufacturer and catalog number in parentheses): Aurora B T232 (1:100, Rockland Immunochemicals, Inc., 600-401-677), Sgo1 (F-8, 1:100, Santa Cruz Biotechnology Inc., sc-393993), PLK1 (E-2, 1:100, Santa Cruz Biotechnology Inc., sc-55504), PLK4 (6H5, 1:500, Millipore, MABC544), SAS-6 (91.390.21, 1:100, Santa Cruz Biotechnology Inc., sc-81431), STIL (1:500, Abcam, ab89314), C-NAP1 (1:100, Proteintech, 14498-1-AP), VPS4B (UT292, 1:50, Sigma-Aldrich, ABS1656), ALIX (3A9, 1:100, Thermo Scientific Pierce Antibodies, MA1-83977), lamin B1 (EPR8985(B), 1:100, Abcam, ab194106), pericentrin (1:100, Proteintech, 22271-1-AP), β-tubulin E7 monoclonal antibody (1:100, Developmental Studies Hybridoma Bank USA, E7), γ-tubulin (GTU-88, 1:100, Sigma-Aldrich, T6557), c-Myc(9E10, 1:1000, Santa Cruz Biotechnology Inc., sc-40), centrin(1:100, Proteintech, 12794-1-AP), purified PLK1 pT539 (1:400, AbClon), purified PLK1 pS529 (1:200, AbClon), phospho-H2AX (1:200, Cell Signaling), phospho-MPM2 (1:1000, Millipore, 05-368), Alexa Fluor® 488 goat anti-rabbit IgG (1:100, Thermo Scientific Pierce Antibodies, A11034), Alexa Fluor® 594 goat anti-mouse IgG (1:100, Thermo Scientific Pierce Antibodies, A11032), and Alexa Fluor® 594 goat anti-rabbit IgG (1:100, Thermo Scientific Pierce Antibodies, A11037).

### Cell culture and transfection

HeLa and RPE1 cells were obtained from the American Type Culture Collection (ATCC) and cultured in Dulbecco’s modified Eagle’s medium (DMEM, WelGENE Inc.) supplemented with 10% fetal bovine serum (FBS, Invitrogen), 100 units/ml penicillin and 100 μg/ml streptomycin (Invitrogen). The cells were maintained at 37 °C in a humidified atmosphere containing 5% CO2. All cell lines tested negative for mycoplasma contamination by PCR. The Fingerprinting of all cell lines by ‘AmplFLSTR identifiler PCR Amplification kit’ was tested in Korean Cell Line Bank. siRNAs were transfected into HeLa cells using DharmaFect 1 (Dharmacon, Inc.). DNA transfection was performed using Lipofectamine 2000 (Invitrogen, USA) in accordance with the manufacturer’s instructions.

### Cell treatments

To generate DNA damage, HeLa cells were treated with 0.5 µM B[a]P (Sigma) and 2.5 µM B[a]P-7,8-diol-9,10-epoxide (Sigma) for 48 h or 0.5 µM etoposide (Sigma) for 24 h. For the inhibition experiments, cells were incubated with the ATR inhibitor NU6027 (2 µM) (Sigma), the ATM inhibitor KU55933 (20 nM) (Sigma), (Enzo Life Sciences), the Chk1 inhibitor UCN-01 (300 nM) (Sigma), the Chk2 inhibitor (1 µM), and the DNA-PK inhibitor (0.5 µM) for 24 h. For synchronization in G2, cells were treated with the Cdk1 inhibitor RO3306 (1 µM) for 24 h. For inhibition of mitotic kinases, cells were treated with the Cdk1 inhibitor RO3306 (1 µM) for 5 h, the Plk1 inhibitor BI2536 (1 µM) for 1 h, the Aurora A inhibitor VX680 (5 µM) for 1 h, and the Aurora B inhibitor Hesperadin (2 µM) (Selleckem) for 5 h. Mitotic arrest was achieved by the treatment of 100 ng/ml nocodazole for 16 h.

### siRNAs and primers

The control siRNA was 5’-CGTACGCGGAATACTTCGATT-3’. The following siRNA sequences were used: siHornerin-A: 5‘-GCAACAUGGUUCUACAUCAUU-3‘, siHornerin-B: 5‘-CUGUCUUUGGUCAACAUGAUU-3‘, siSeparase: 5′-CAAGGUUAGAUUUAAUCCUUU-3′, siPlk1: 5′-CCUUGAUGAAGAAGAUCAC-3′, siAhR: 5’-CGUUAGAUGUUCCUCUGUG-3’. Detailed information on the primers used in this study is provided in Supplementary Table S1.

### Immunoprecipitation

Antibodies were conjugated to Affi-Prep Protein A beads (Bio-Rad Laboratories) at a concentration of 0.3 mg/ml. We lysed 1 × 10^7^ cells in 1 ml NP-40 lysis buffer (50 mM HEPES, pH 7.4, 200 mM KCl, 0.3% NP-40, 10% glycerol, 1 mM EGTA, 1 mM MgCl2, 0.5 mM DTT, and 10 µg/ml each of leupeptin, pepstatin, and chymostatin). To preserve phosphorylation, lysis buffer was supplemented with 1 mM NaVO4 and 0.5 µM microcystin LR (Alexis Biochemicals). Corresponding lysates were centrifuged, incubated with protein A beads coupled with preimmune rabbit IgG at 4 °C for 1 h, and then incubated with protein A beads coupled with specific antibodies at 4 °C overnight. The beads were washed 5x with lysis buffer, boiled in Laemmli sample buffer for 3 min and resolved via SDS–polyacrylamide gel electrophoresis (PAGE).

### Immunofluorescence and live cell imaging

HeLa cells on coverslips were fixed with methanol at −20 °C for 30 min. Alternatively, soluble proteins were extracted from the cells with BRB80-T buffer (80 mM PIPES, pH 6.8, 1 mM MgCl2, 5 mM EGTA and 0.5% Triton X-100) and then fixed with 4% paraformaldehyde for 15 min at room temperature. Fixed cells were permeabilized and blocked with PBS-BT (1 × PBS, 3% BSA, and 0.1% Triton X-100) for 30 min at room temperature. Coverslips were then incubated with primary and secondary antibodies diluted in PBS-BT. Images were acquired using ZEN2 software (Carl Zeiss, Germany) under a Zeiss Axiovert 200 M microscope with a 1.4 NA plan-Apo 100× oil immersion lens and an HRm CCD camera. Deconvoluted images were obtained and analyzed using AutoDeblur v9.1 and AutoVisualizer v9.1 (AutoQuant Imaging). The insets show single focal planes of the boxed regions. All images in a panel were acquired under a constant exposure time for all channels. To eliminate the size difference in each marked region, the average intensity was obtained from the selected area. Upon background subtraction, the average intensities of the desired channel were normalized against the average intensity of the corresponding markers or subtracted from the average background intensity value. For the fluorescence intensity plot, all intensities were normalized to those of the control and plotted as relative intensities.

For time-lapse microscopy, HeLa cells stably expressing GFP-H2B or GFP-centrin 2 were cultured in Leibovitz’s L-15 medium (Invitrogen) supplemented with 10% FBS (Invitrogen) and 2 mM L-glutamine (Invitrogen). The cells were placed in a sealed growth chamber that was heated to 37 °C and were observed using a Zeiss Axiovert 200 M microscope with a 20× lens. Images were acquired every three minutes for 5 h using AxioVision 4.8.2 (Carl Zeiss).

### Bacterially expressed proteins

pET-28-vector encoded, His6-tagged Plk1 WT, S529A, T539A, 3 A, 3D, or Sgo1 were expressed individually in E. coli BL21 DE3 (Novagen). We lysed bacteria in lysis buffer (50 mM Na2HPO4, 100 mM NaCl, 10% glycerol, 10 mM imidazole), added Ni2 + -NTA-agarose (Qiagen), and washed beads with washing buffer (50 mM Na2HPO4, 100 mM NaCl, 10% glycerol, 20 mM imidazole). Corresponding proteins were purified from Ni2 + -NTA-agarose (Qiagen) according to standard procedures.

### Pull-down assay

For the in vitro pull-down assay, 1 µg of each protein (His-Plk1 WT, 3 A, 3D, or His-Sgo1) was incubated with 20 µl of Ni2 + -NTA-agarose beads (Qiagen) for 1 h at 4 °C. After three washes with washing buffer (50 mM Na2HPO4, 100 mM NaCl, 10% glycerol, 20 mM imidazole), the protein-bead complex was incubated with the corresponding cell lysates (500 µg of total protein) for 1 h at 4 °C. After being washed with washing buffer three times, the beads were denatured by the addition of Laemmli sample buffer and boiling for 5 min at 95 °C. Samples were analyzed by immunoblotting.

### In vitro kinase assay

For the Chk1 kinase assay, 200 ng of human recombinant Chk1 kinase (SignalChem) and Aurora A (purified from asynchronous *sf9* cells) were incubated with 1 μg of His-Plk1 WT, T210A, S526A, S529A, or T539A mutant and 10 μM ATP in 50 μl of kinase buffer (25 mM Tris-HCl (pH 7.5), 2 mM DTT, 10 mM MgCl2, 5 mM β-glycerophosphate, and 0.1 mM Na3VO4) for 30 min at 37 °C. Incorporation of phosphate into His-Plk1 was visualized by SDS-PAGE and immunoblotting with specific phospho-antibodies.

### Tandem purification and proteomics

HeLa S3 cells expressing GFP-S-Plk1 were generated as described previously [78]. The cells were lysed in 1 ml of NP-40 lysis buffer (50 mM Hepes, pH 7.4, 200 mM KCl, 0.3% NP-40, 10% glycerol, 1 mM EGTA, 1 mM MgCl2, 0.5 mM DTT, 1 mM NaVO4, 0.5 μM microcystin LR, 5 mM C4H7NaO2, and 10 μg/ml each of leupeptin, pepstatin, and chymostatin). Lysates were centrifuged and incubated with protein A beads coupled with anti-GFP antibodies at 4 °C overnight. Antibody beads were recovered by centrifugation, washed five times with lysis buffer in the presence of 500 mM KCl, washed twice with lysis buffer W/O protease inhibitors, and treated with TEV protease overnight to release S-Plk1 from protein A beads and GFP antibodies. Protease inhibitors were added to the supernatant and incubated with S-protein agarose beads (Sigma) for 3 h at 4 °C. The beads were washed 5x with lysis buffer in the presence of 0.5 mM DTT and incubated with urea elution buffer (50 mM Tris pH 8.5, 8 M urea) for 30 min at room temperature. Purified protein complexes were analyzed by mass spectrometry as described previously [78].

### In situ PLA assay

Cells transfected with plasmid were blocked with Duolink blocking solution (Signa Aldrich) for 60 min at 37 °C. Afterward, the cells were incubated with Myc primary antibody (1:100), Hornerin primary antibody (1:100) for 30 min at 37 °C. The cells were washed in wash buffer A and incubated with PLUS and MINUS PLA probes (1:5) for 60 min at 37 °C. The cells were washed in wash buffer A and then incubated with the ligase solution for 30 min at 37 °C. The cells were again washed in wash buffer A and incubated with the polymerase solution for 100 min at 37 °C. Finally the cells were washed in wash buffer B for 20 min and then 0.01x buffer B for 4 min. After DAPI was added, the cells were mounted and imaged.

### Centrosome preparation

Growing cells were incubated with culture media containing 10 mM nocodazole and 10 mM cytochalasin D for 1 h at 37 °C. Cells were harvested and lysed in a solution (1 mM HEPES pH 7.2, 0.5% NP-40, 0.5 mM MgCl_2_, 0.1% β-mercaptoethanol, 1 mM PMSF and protease inhibitors (aprotinin, leupeptin, pepstatin at 1 μg/mL)). Swollen nuclei and chromatin aggregates were removed by centrifugation at 3500 rpm for 10 min, and the supernatant was filtered through a nylon mesh. After adding 10 mM HEPES and 1 μg/mL DNase I, lysate was loaded on the 50% sucrose solution and centrosomes were sedimented on a sucrose cushion by centrifugation at 7500 rpm for 30 min. After removing 1/3 of the initial volume of the supernatant from the top, resuspended supernatant was loaded onto a sucrose gradient consisting of 3 ml of 70%, 3 ml of 50%, and 3 ml of 40% sucrose solution and centrifuged at 25000 rpm for 1 h. Fourteen fractions with 500 μl per fraction were collected from the bottom.

### Colony formation assay

The cells were seeded into dishes at a density of 500 cells per dish and incubated for 2 weeks. The cell culture medium was refreshed every 3 days. After 2 weeks, the cells were stained with crystal violet (0.5 mg/ml, Santa Cruz Biotechnology Inc.) for 30 min on a shaker after washing with PBS. After 30 min, the cells were washed with PBS several times. The colonies were photographed and cell colonies with >50 cells or more were counted.

### TCGA RNA-seq data

All TCGA RNA-seq data were acquired from the Human Protein Atlas website (https://www.proteinatlas.org/). Based on the FPKM (number fragments per kilobase of exon per million reads) value of each gene, patients were classified into two expression groups and the correlation between expression level and patient survival was examined. The survival outcomes of the two groups were compared by Kaplan–Meier survival analysis and log-rank tests. All statistical analyses were performed using IBM SPSS Statistics version 20.0 (IBM Inc., Armonk, NY, USA). All tests were two-sided, and a *p-*value of 0.05 was used as the cutoff value to determine statistical significance.

### Statistical analysis

Statistical analyses were performed with GraphPad Prism software using two-way ANOVA with Bonferroni’s multiple comparisons test or a two-tailed *t* test as indicated in the figure legends. The error bars represent the standard errors of the mean (SEMs) of 3 independent experiments. A *p-*value of <0.01 (two-tailed) was considered statistically significant. For phenotype analysis in mitotic cell, we randomly analyzed 30 cells from three independent experiments. Because the percentage of mitotic cells in culture dish is about 1~2%, 10 cells are sufficient for each experiment. For quantitative analysis of immunostaining, the investigators were blinded to sample allocation.

## Supplementary information

Supplementary Figures and Table

Supplementary Legends

Original Data File

Video-1

Video-2

Video-3

Video-4

Video-5

Video-6

Video-7

Video-8

Video-9

## Data Availability

All relevant data are readily available from the authors.
