# Supplementary material for: Hornerin mediates phosphorylation of the polo-box domain in Plk1 by Chk1 to induce death in mitosis
Source: Cell Death Differ. 2023 Aug 18;30(9):2151–66. doi: 10.1038/s41418-023-01208-y (PMC10482915; doi:10.1038/s41418-023-01208-y)
Supplement: Supplementary file 1 — Supplementary Figures and Table [file 41418_2023_1208_MOESM1_ESM.pdf]

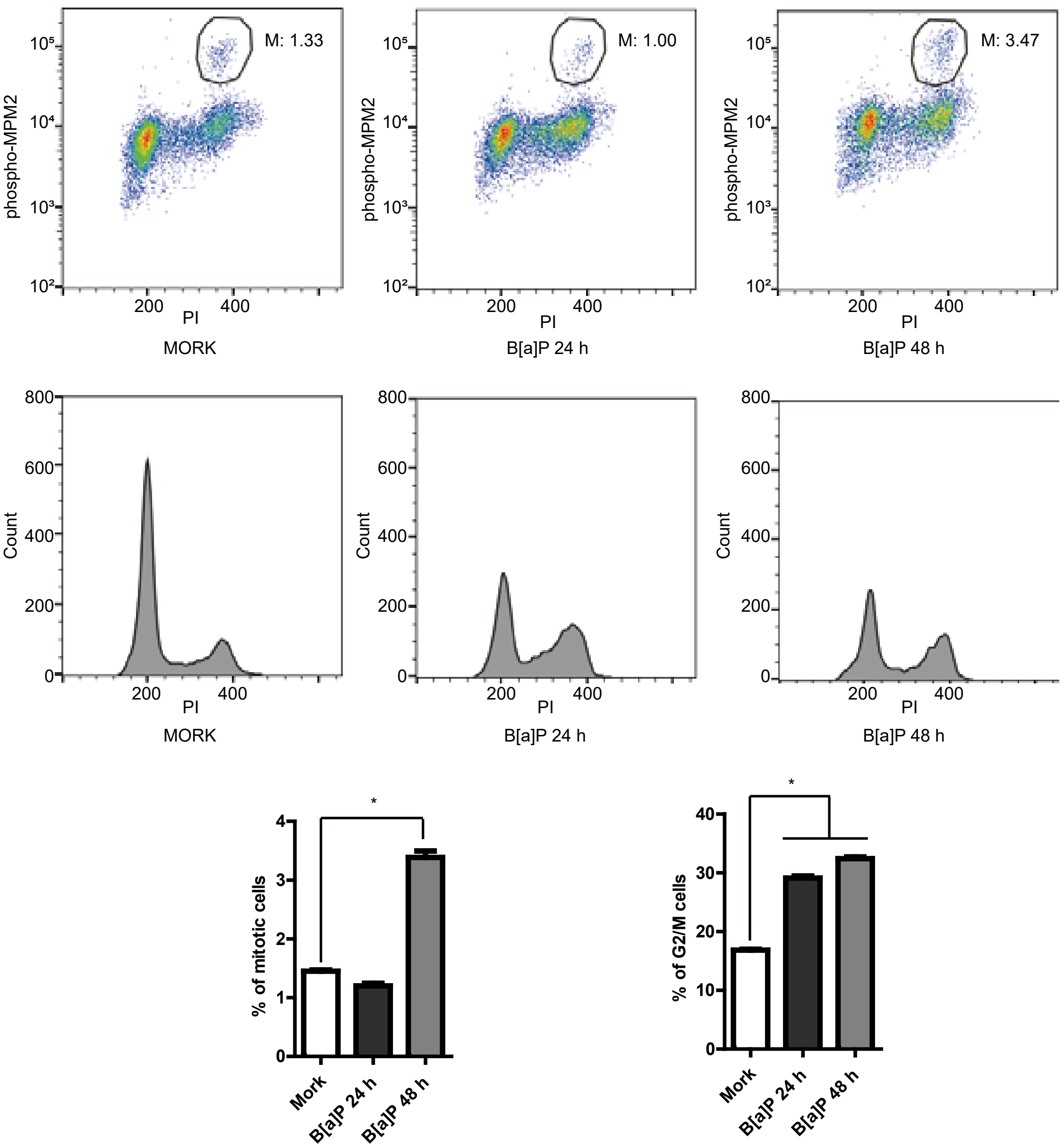
M: 1.33
M: 1.00
M: 3.47
phospho-MPM2
PI
MORK
B[a]P 24 h
B[a]P 48 h
Count
% of mitotic cells
% of G2/M cells
Mork
*

Figure S1.

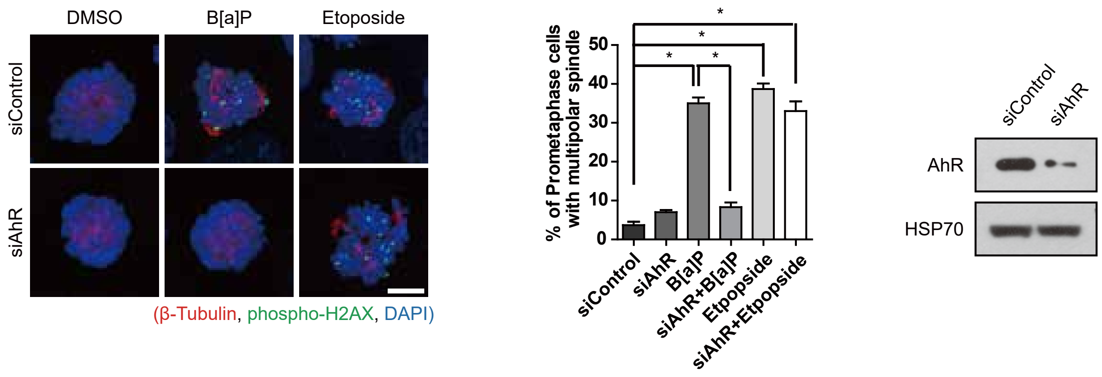
DMSO
B[a]P
Etoposide
siControl
siAhR
(β-Tubulin, phospho-H2AX, DAPI)
% of Prometaphase cells with multipolar spindle
50
40
30
20
10
0
*
*
*
*
siControl
siAhR
B[a]P
siAhR+B[a]P
Etpopside
siAhR+Etpopside
siControl
siAhR
AhR
HSP70

Figure S2.

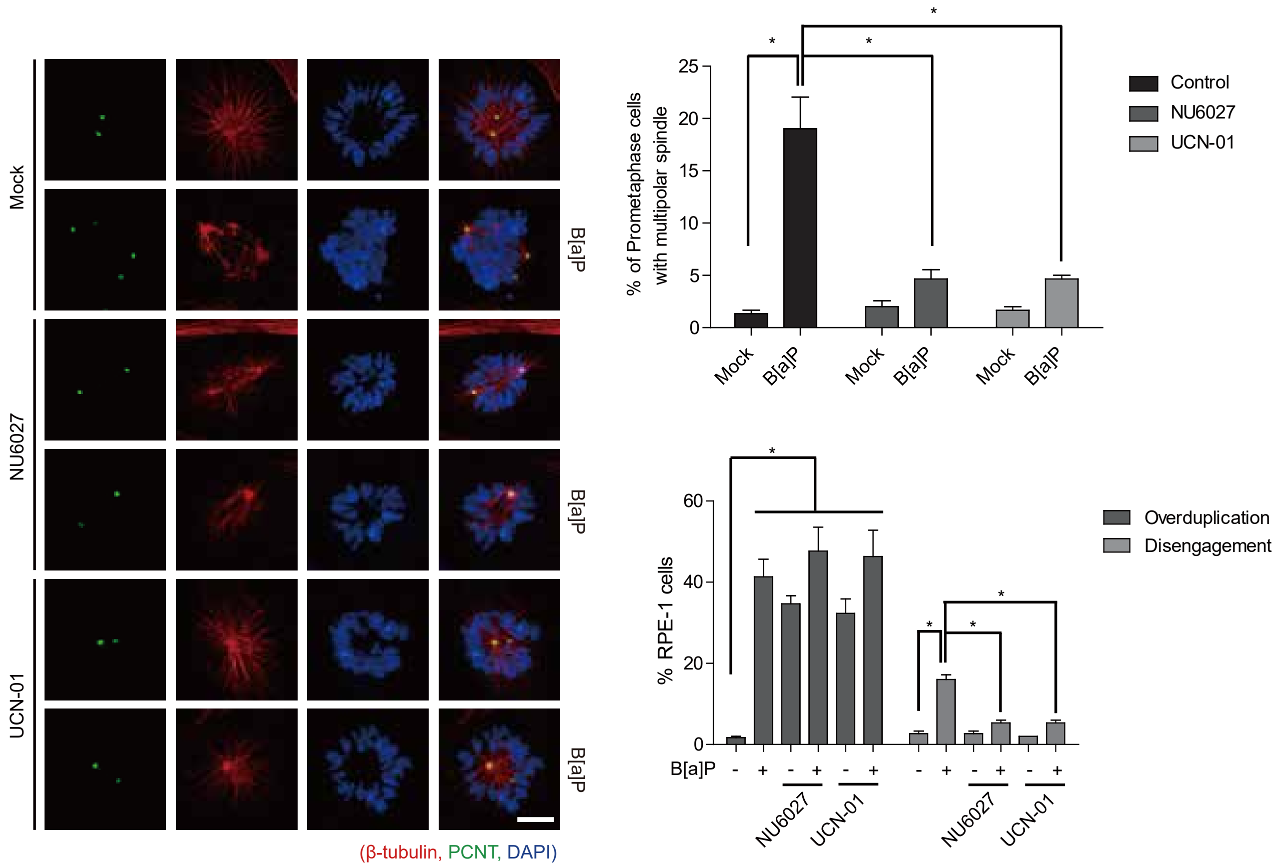

Mock
NU6027
UCN-01
B[a]P
B[a]P
B[a]P
(β-tubulin, PCNT, DAPI)
% of Prometaphase cells with multipolar spindle
25
20
15
10
5
0
Mock
B[a]P
Mock
B[a]P
Mock
B[a]P
*
*
*
Control
NU6027
UCN-01
% RPE-1 cells
60
40
20
0
B[a]P
- + - + - + - + - + - +
NU6027
UCN-01
NU6027
UCN-01
*
*
*
*
Overduplication
Disengagement

Figure S3.

A

Phospho-peptides of Plk1

KpT$_{210}$LCGpT$_{214}$PNYIAPEVLSKK (XCorr: 1.44099)
KTLCGTPNYIAPEVLpS$_{224}$KK (XCorr: 1.607579)
LPITCLTIPPRFSIAPpS$_{330}$SLDPSNR (XCorr: 1.061873)
pY$_{481}$FRNpY$_{485}$MpS$_{487}$EHLLKAGANITPR (XCorr: 2.188277)
SAIILHLpS$_{526}$NGpS$_{529}$VQINFFQDHTK (XCorr: 1.126777)
pS$_{519}$AIILHLSNGSVQINFFQDHpT$_{539}$KLILcPLmAAVTyIDEKR (XCorr: 2.827393)
LLSpS$_{593}$RpS$_{595}$ApS$_{597}$NRLK (XCorr: 1.129912)

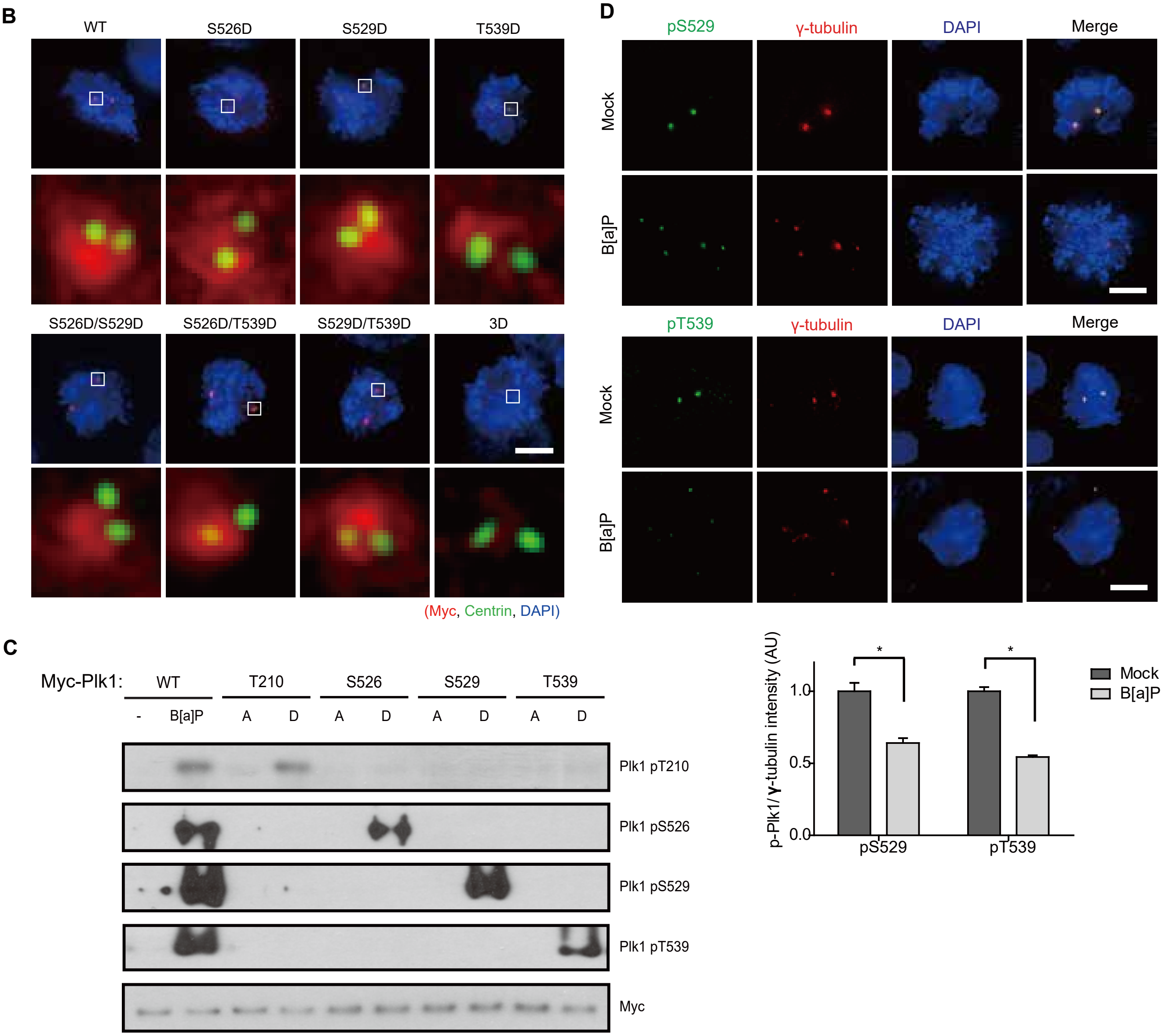

Figure S4.

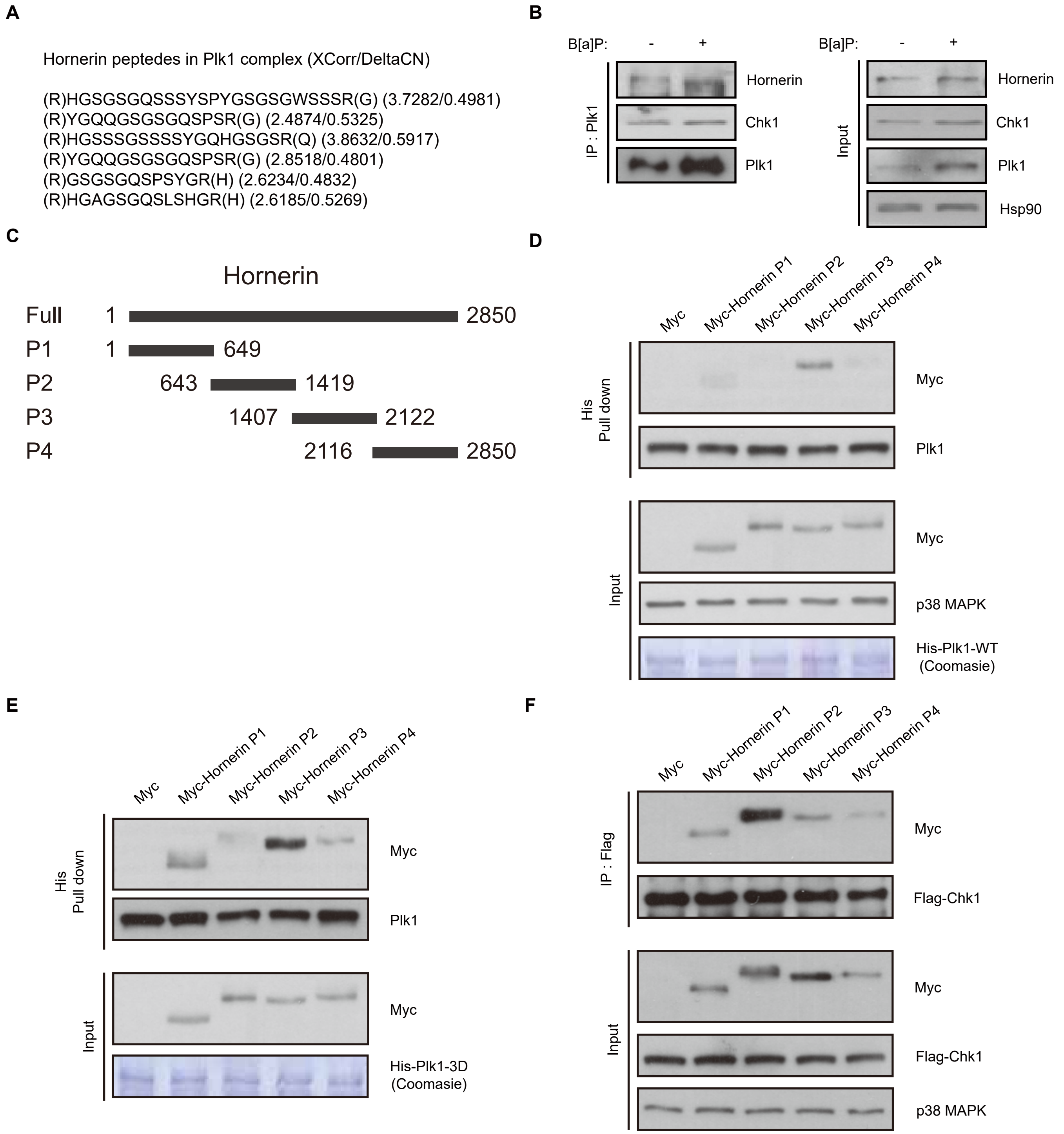
A
Hornerin peptedes in Plk1 complex (XCorr/DeltaCN)
(R)HGSGSGQSSSYSPYGSGSGWSSSR(G) (3.7282/0.4981)
(R)YGQQGSGSGQSPSR(G) (2.4874/0.5325)
(R)HGSSSGSSSSYGQHGSGSR(Q) (3.8632/0.5917)
(R)YGQQGSGSGQSPSR(G) (2.8518/0.4801)
(R)GSGSGQSPSYGR(H) (2.6234/0.4832)
(R)HGAGSGQSLSHGR(H) (2.6185/0.5269)
B
B[a]P: - +
IP : Plk1
Hornerin
Chk1
Plk1
B[a]P: - +
Input
Hornerin
Chk1
Plk1
Hsp90
C
Hornerin
Full 1 2850
P1 1 649
P2 643 1419
P3 1407 2122
P4 2116 2850
D
Myc
Myc-Hornerin P1
Myc-Hornerin P2
Myc-Hornerin P3
Myc-Hornerin P4
His Pull down
Myc
Plk1
Input
Myc
p38 MAPK
His-Plk1-WT (Coomasie)
E
Myc
Myc-Hornerin P1
Myc-Hornerin P2
Myc-Hornerin P3
Myc-Hornerin P4
His Pull down
Myc
Plk1
Input
Myc
His-Plk1-3D (Coomasie)
F
Myc
Myc-Hornerin P1
Myc-Hornerin P2
Myc-Hornerin P3
Myc-Hornerin P4
IP : Flag
Myc
Flag-Chk1
Input
Myc
Flag-Chk1
p38 MAPK

Figure S5.

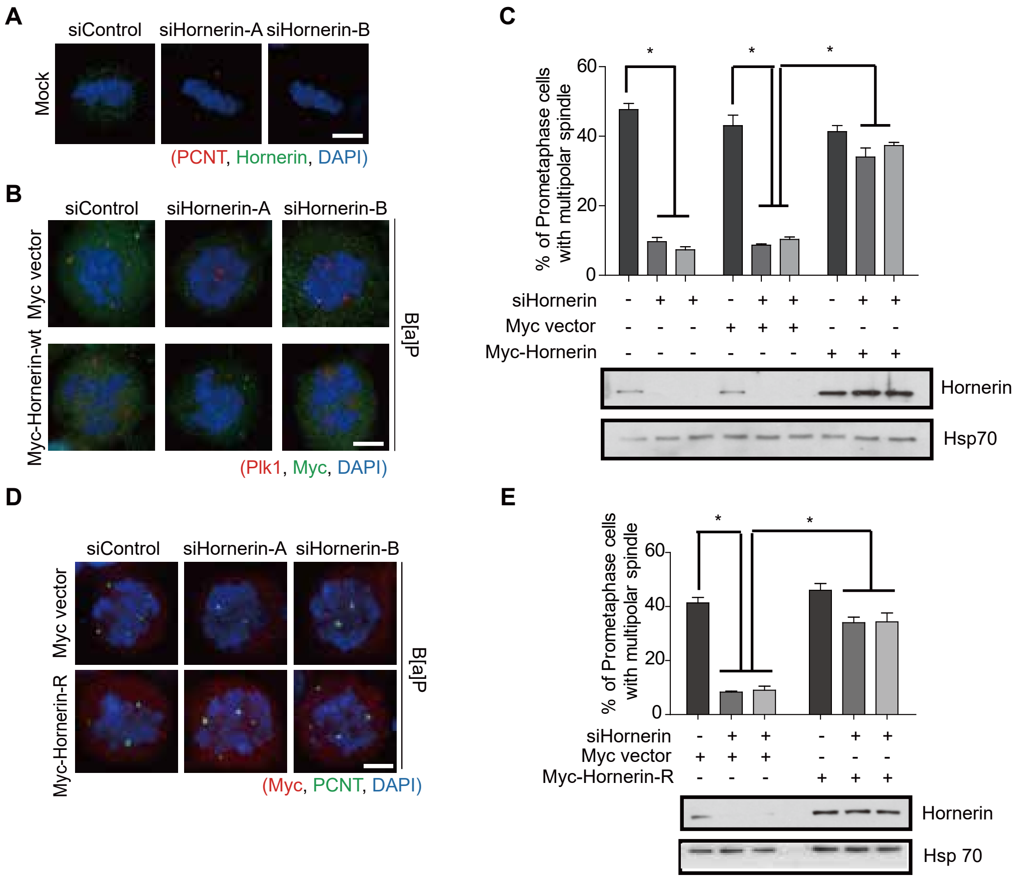
A
siControl
siHornerin-A
siHornerin-B
Mock
(PCNT, Hornerin, DAPI)
B
siControl
siHornerin-A
siHornerin-B
Myc vector
Myc-Hornerin-wt
B[a]P
(Plk1, Myc, DAPI)
C
% of Prometaphase cells with multipolar spindle
60
40
20
0
*
*
*
siHornerin
Myc vector
Myc-Hornerin
Hornerin
Hsp70
D
siControl
siHornerin-A
siHornerin-B
Myc vector
Myc-Hornerin-R
B[a]P
(Myc, PCNT, DAPI)
E
% of Prometaphase cells with multipolar spindle
60
40
20
0
*
*
siHornerin
Myc vector
Myc-Hornerin-R
Hornerin
Hsp 70

Figure S6.

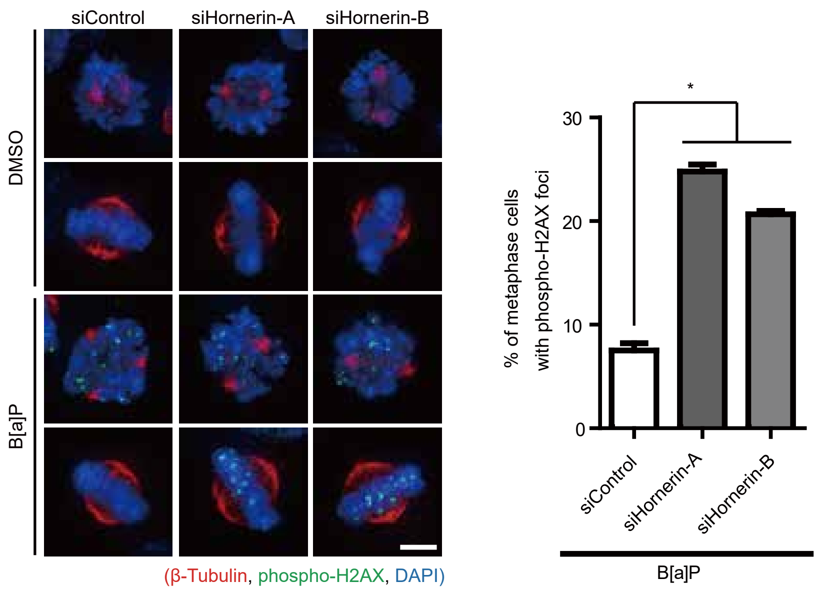
siControl
siHornerin-A
siHornerin-B
DMSO
B[a]P
(β-Tubulin, phospho-H2AX, DAPI)
*
30
20
10
0
% of metaphase cells with phospho-H2AX foci
siControl
siHornerin-A
siHornerin-B
B[a]P

Figure S7.

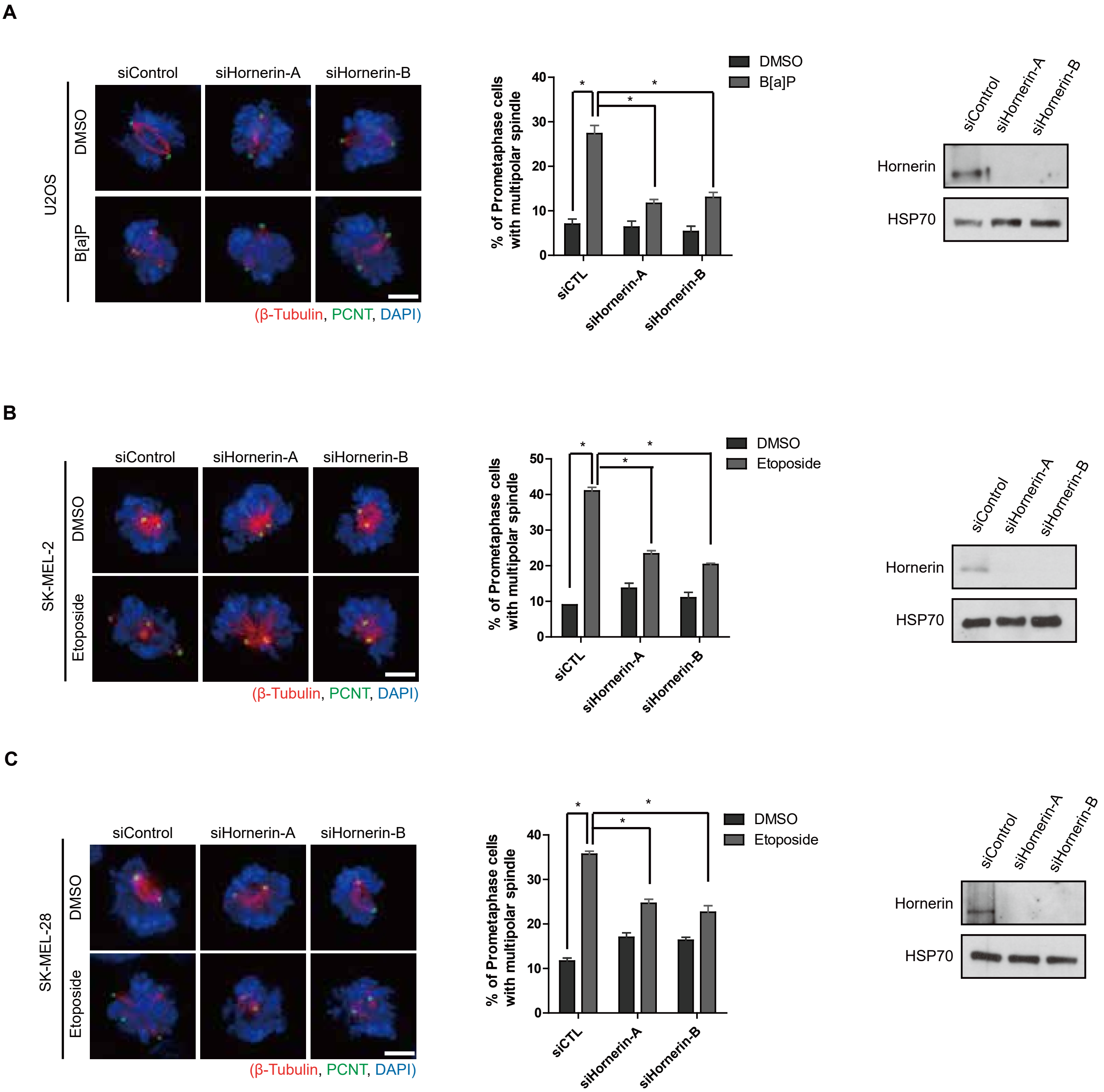
A
siControl
siHornerin-A
siHornerin-B
U2OS
DMSO
B[a]P
(β-Tubulin, PCNT, DAPI)
% of Prometaphase cells with multipolar spindle
40
30
20
10
0
*
*
*
DMSO
B[a]P
siCTL
siHornerin-A
siHornerin-B
siControl
siHornerin-A
siHornerin-B
Hornerin
HSP70
B
siControl
siHornerin-A
siHornerin-B
SK-MEL-2
DMSO
Etoposide
(β-Tubulin, PCNT, DAPI)
% of Prometaphase cells with multipolar spindle
50
40
30
20
10
0
*
*
*
DMSO
Etoposide
siCTL
siHornerin-A
siHornerin-B
siControl
siHornerin-A
siHornerin-B
Hornerin
HSP70
C
siControl
siHornerin-A
siHornerin-B
SK-MEL-28
DMSO
Etoposide
(β-Tubulin, PCNT, DAPI)
% of Prometaphase cells with multipolar spindle
40
30
20
10
0
*
*
*
DMSO
Etoposide
siCTL
siHornerin-A
siHornerin-B
siControl
siHornerin-A
siHornerin-B
Hornerin
HSP70

Figure S8.

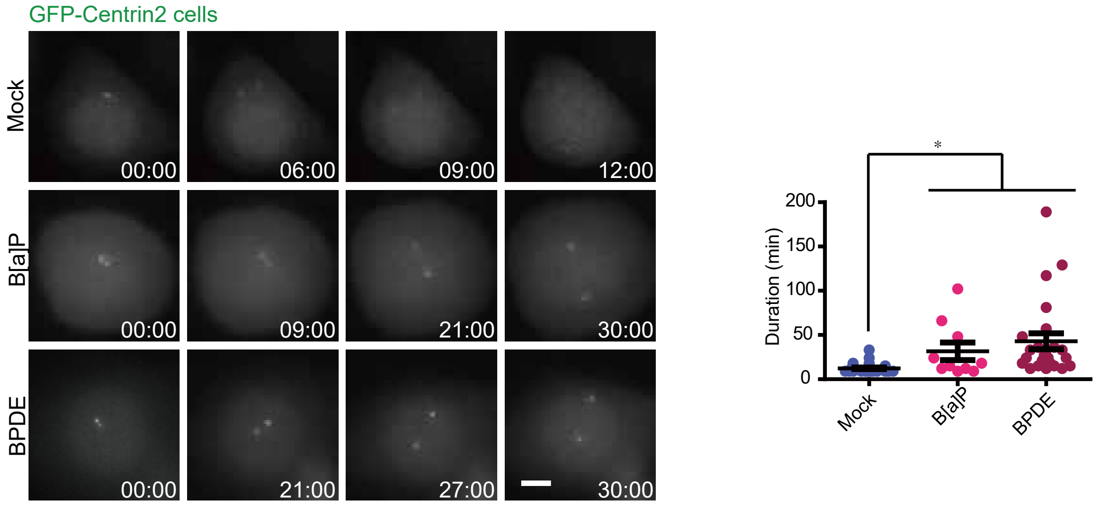
GFP-Centrin2 cells
Mock
B[a]P
BPDE
00:00
06:00
09:00
12:00
00:00
09:00
21:00
30:00
00:00
21:00
27:00
30:00
*
Duration (min)
200
150
100
50
0
Mock
B[a]P
BPDE

Figure S9.

A

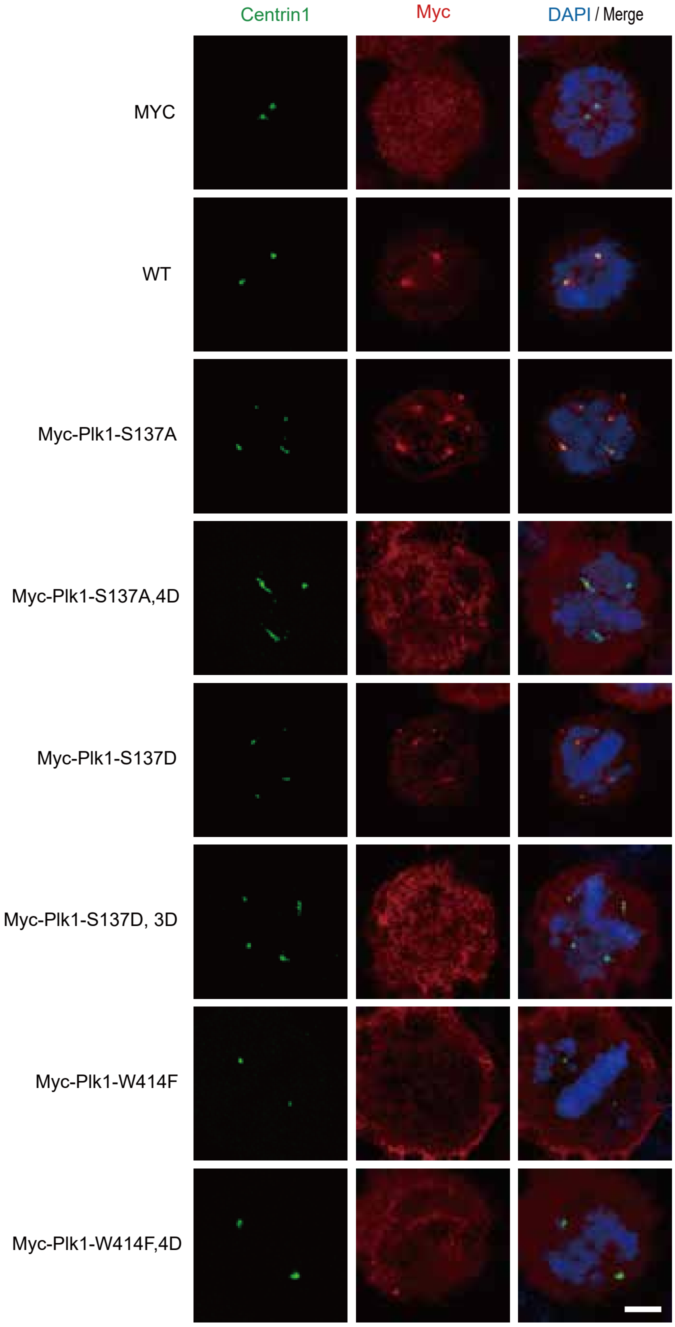

B

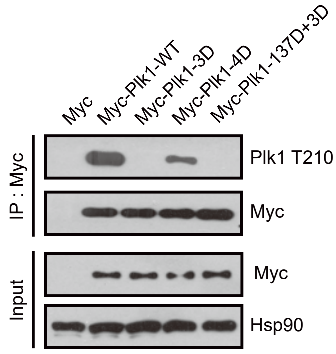

Figure S10.

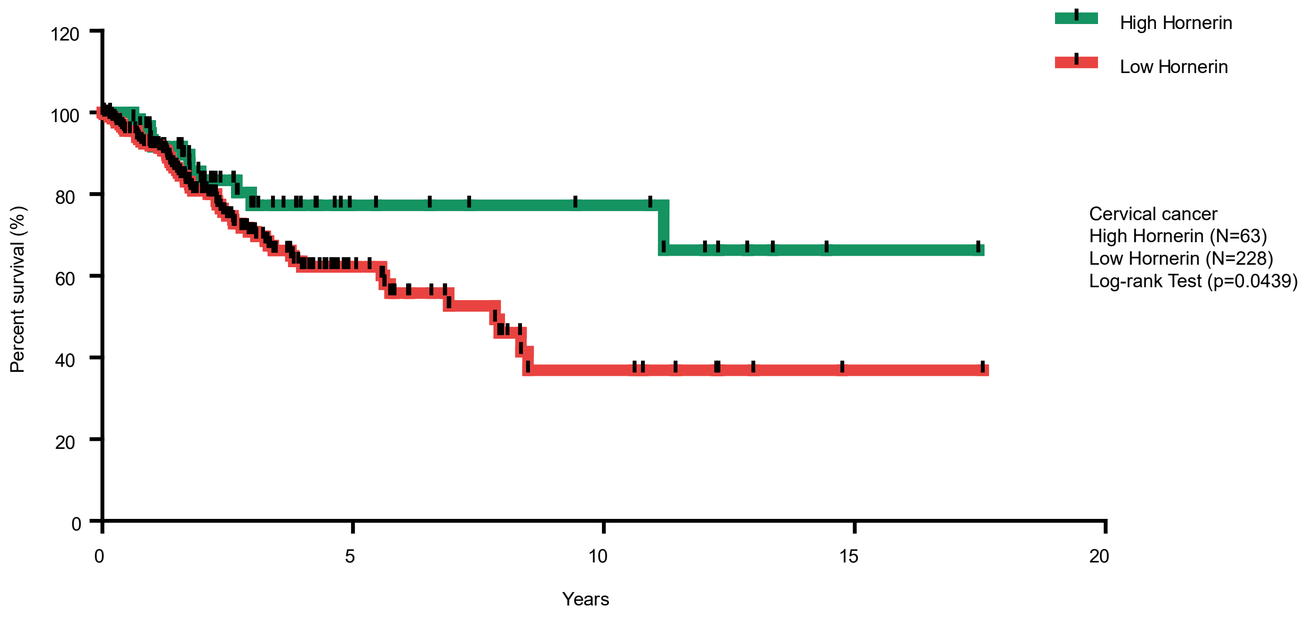
High Hornerin
Low Hornerin
120
100
80
60
40
20
0
Percent survival (%)
0
5
10
15
20
Years
Cervical cancer
High Hornerin (N=63)
Low Hornerin (N=228)
Log-rank Test (p=0.0439)

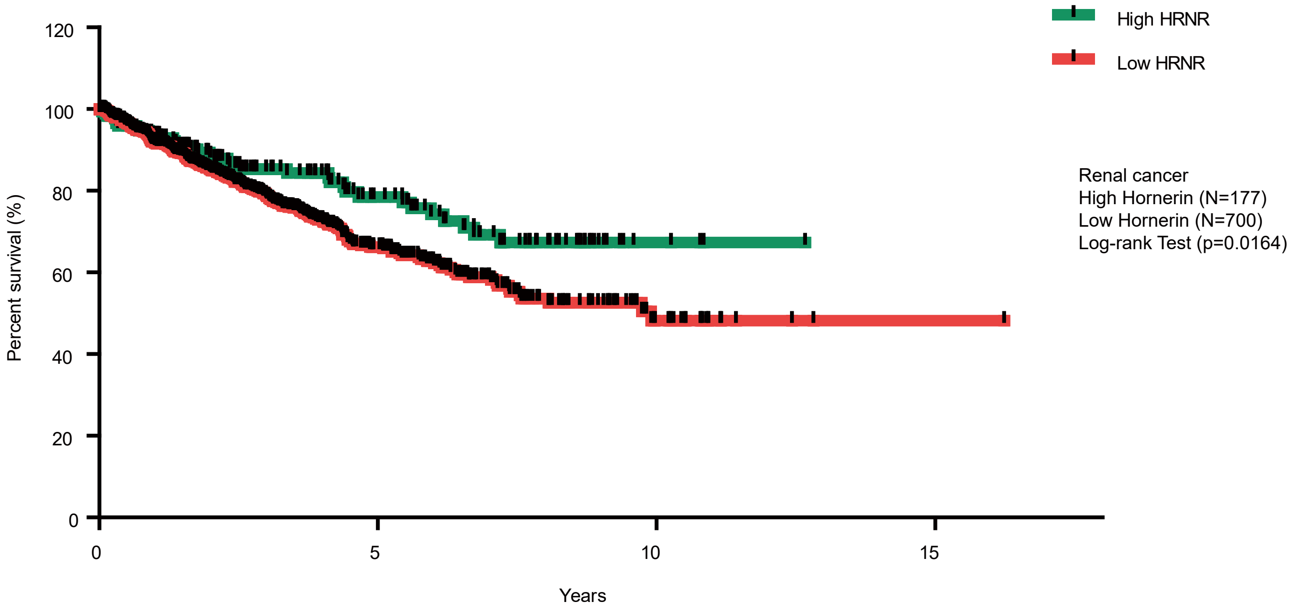
High HRNR
Low HRNR
120
100
80
60
40
20
0
Percent survival (%)
0
5
10
15
Years
Renal cancer
High Hornerin (N=177)
Low Hornerin (N=700)
Log-rank Test (p=0.0164)

Figure S11.

Table S1. Primer list

Primer	Sequence
Plk1-Forward	ATATGGCCGGCCCATGAGTGCTGCAGTGACT
Plk1-Reverse	ATATGGCGCGCCTTAGGAGGCCTTGAGACG
Plk1-S526D-Forward	ATCATCCTGCACCTCGACAACGGCAGCGTG
Plk1-S526D-Reverse	CACGCTGCCGTTGTCGAGGTGCAGGATGAT
Plk1-S529D-Forward	CACCTCAGCAACGGCGACGTGCAGATCAAC
Plk1-S529D-Reverse	GTTGATCTGCACGTCGCCGTTGCTGAGGTG
Plk1-S539D-Forward	TTCTTCCAGGATCACGACAAGCTCATCTTG
Plk1-S539D-Reverse	CAAGATGAGCTTGTCGTGATCCTGGAAGAA
Plk1-S526/529D-Forward	CTGCACCTCGACAACGGCGACGTGCAGATC
Plk1-S526/529D-Reverse	GATCTGCACGTCGCCGTTGTCGAGGTGCAG
Plk1-S526A-Forward	ATCATCCTGCACCTCGCCAACGGCAGCGTG
Plk1-S526A-Reverse	CACGCTGCCGTTGGCGAGGTGCAGGATGAT
Plk1-S529A-Forward	CACCTCAGCAACGGCGCCGTGCAGATCAAC
Plk1-S529A-Reverse	GTTGATCTGCACGGCGCCGTTGCTGAGGTG
Plk1-S539A-Forward	TTCTTCCAGGATCACGCCAAGCTCATCTTG
Plk1-S539A-Reverse	CAAGATGAGCTTGGCGTGATCCTGGAAGAA
Plk1-S526/529A-Forward	CTGCACCTCGCCAACGGCGCCGTGCAGATC
Plk1-S526/529A-Reverse	GATCTGCACGGCGCCGTTGGCGAGGTGCAG
Sgo1-Forward	ATATGGCCGGCCCATGGCCAAGGAAAGATGC
Sgo1-Reverse	ATATGGCGCGCCTTGTATTTGTTTCATACT
Hornerin-P1-Forward	CTTGGTACCATGGAGCAGAAACTCATCTCT
Hornerin-P1-Reverse	GCCGAATTCTCAATATCTGCTGGACTGGGAAGA
Hornerin-P2-Forward	GGCGGTACCTCTTCCCAGTCCAGCAGATA
Hornerin-P2-Reverse	GTGGAATTCTCAGCTGCCGTGCTGTGTACAGCC
Hornerin-P3-Forward	CACGGTACCTCCAGCTGGCAGAGCAGCGGC
Hornerin-P3-Reverse	GCCGAATTCTCACTGAGAGTAGGAAGAGGACTGG
Hornerin-P4-Forward	TCCGGTACCCAGTCCTCTTCCTACTCTCAG
Hornerin-P4-Reverse	GCAGAATTCTCACTGGTAGAAATAACATCT
Hornerin-R-Forward	GGGCAACATGGATCTACTTCAGGACAG
Hornerin-R-Reverse	CTGTCCTGAAGTAGATCCATGTTGCCC
