# Supplementary material for: Hornerin mediates phosphorylation of the polo-box domain in Plk1 by Chk1 to induce death in mitosis
Source: Cell Death Differ. 2023 Aug 18;30(9):2151–66. doi: 10.1038/s41418-023-01208-y (PMC10482915; doi:10.1038/s41418-023-01208-y)
Supplement: Supplementary file 2 — Supplementary Legends [file 41418_2023_1208_MOESM2_ESM.docx]

**SUPPLEMENTARY FIGURE LEGENDS**

**Figure S1.** DNA damage causes G2/M arrest. HeLa cells treated with 0.5 µM B[a]P for 24 h or 48 h were stained with phospho-MPM2 antibody and propidium iodide (PI) for mitotic phosphoproteins and DNA, respectively. The cells were analyzed by FACS (n = 60000 cells from three independent experiments). M, Mitotic population, Error bars, SEM. *, p < 0.01 (two-tailed *t* test).

**Figure S2**. AhR is responsible for DNA damage caused by B[a]P. siRNA-transfected HeLa cells were treated with 0.5 µM B[a]P for 48 h or 0.5 µM etoposide for 24 h and analyzed by immunostaining (n = 300 cells from three independent experiments). Scale bar, 5 µm. Error bars, SEM. *, p < 0.01 (two-tailed *t* test).

**Figure S3**. DDR induces premature centriole disengagement in RPE-1 cells. Twenty-four hours after treatment with the indicated inhibitors, RPE-1 cells were treated with 0.5 µM B[a]P for 48 h or 0.5 µM etoposide for 24 h, and multipolar spindles or centriole disengagement were analyzed (n = 300 cells for multipolar spindle or 400 centrioles for centriole disengagement, respectively, from three independent experiments). Scale bar, 5 µm. Error bars, SEM. *, p < 0.01 (two-tailed t test).

**Figure S4**. Phosphorylation and translocation of Plk1 via the DDR. **A** Plk1 complex-derived peptides corresponding to phospho-Plk1 are presented, with the indicated XCorr scores. **B** HeLa cells were transfected with the indicated Plk1 mutants and stained with the indicated antibodies. **C** Twenty-eight hours after transfection with the indicated plasmids, cell lysates were analyzed by immunoblotting. **D** Cells were treated with B[a]P and analyzed by immunostaining (n = 30). AU, arbitrary units, Scale bars, 5 µm. Error bars, SEM. *, p < 0.01 (two-tailed t test).

**Figure S5.** Hornerin forms a complex with Chk1 and Plk1. **A** Peptides of hornerin identified in the Plk1 complex are listed, with XCorr and DeltaCN scores indicated. **B** HeLa cells treated with B[a]P were subjected to IP-immunoblotting. **C** Diagram of wild-type (WT) hornerin and serial deletion mutants. **D,** **E** Lysates from DNA-transfected HeLa cells were incubated with recombinant His-Plk1 WT (D) or 3D mutant (E), and proteins pulled down with Ni-beads were analyzed by immunoblotting. **F** HeLa cells were transfected with the indicated plasmids and analyzed by IP-immunoblotting. Images of uncropped blots are provided as a Supplementary Material file.

**Figure S6.** Hornerin mediates DDR-induced multipolar spindles. **A** Seventy-two hours after siRNA transfection, cells were stained with the indicated antibodies. **B, C** HeLa cells were transfected with siRNA targeting the untranslated region of hornerin. The cells were transfected with Myc-Hornerin, treated with B[a]P, and analyzed by immunostaining (B) and immunoblotting (C). Prometaphase cells with multipolar spindles were counted, and the values were plotted (C, n = 300). **D, E** Twenty-four hours after siRNA transfection, HeLa cells were transfected with siRNA-resistant hornerin (Myc-Hornerin-R). The cells were treated with B[a]P and analyzed by immunostaining (D) and immunoblotting (E). Prometaphase cells with multipolar spindles were counted, and the values were plotted (E, n = 300). Scale bars, 5 µm. Error bars, SEM. *, p < 0.01 (two-tailed t test). Images of uncropped blots are provided as a Supplementary Material file.

**Figure S7**. Depletion of hornerin increases metaphase cells harboring DNA damage. Twenty-four hours after siRNA transfection, HeLa cells were treated with B[a]P and analyzed by immunostaining. Prometaphase cells with phospho-H2AX foci were counted, and the values were plotted (E, n = 300). Scale bar, 5 µm. Error bars, SEM. *, p < 0.01 (two-tailed t test).

**Figure S8**. Hornerin mediates DDR in skin cells and osteosarcoma epithelial cells. **A-C** siRNA-transfected cells were treated with 0.5 µM B[a]P for 48 h or 0.5 µM etoposide for 24 h and analyzed by immunostaining (n = 300 cells from three independent experiments). Scale bars, 5 µm. Error bars, SEM. *, p < 0.01 (two-tailed *t* test).

**Figure S9**. DDR-mediated Plk1 phosphorylation disrupts centrosome maturation. HeLa/GFP-centrin2 cells were treated with B[a]P or BPDE for 48 h and imaged for GFP-centrin2 by time lapse. The duration of centrosome separation was determined and plotted (n = 30). AU, arbitrary units, Scale bars, 5 µm. Error bars, SEM. *, p < 0.01 (two-tailed t test).

**Figure S10.** The correlation of Plk1 phosphorylation and localization at centrosomes. **A, B** HeLa cells were transfected with myc-Plk1 WT or mutants and analyzed by immunostaining (A) and immunoblotting (B). Scale bar, 5 µm.

**Figure S11.** The survival curves of patients stratified according to the mRNA expression of hornerin in the TCGA database. Log-rank P value for Kaplan-Meier plot showing results from analysis of correlation between mRNA expression level and patient survival.

**TABLE LEGENDS**

**Table S1**. Primer list

**SUPPLEMENTARY MOVIE LEGENDS**

**Movie 1**. Fluorescence time-lapse microscopy of a siControl HeLa/GFP-tubulin cells during

mitosis. Images were captured every 3 min and displayed at 10 frames/s.

**Movie 2**. Fluorescence time-lapse microscopy of B[a]P-treated HeLa/GFP-tubulin cells.

B[a]P-treatment resulted in death in mitosis.

**Movie 3**. Fluorescence time-lapse microscopy of B[a]P-treated HeLa/GFP-tubulin cells.

B[a]P-treatment resulted in regression.

**Movie 4**. Fluorescence time-lapse microscopy of B[a]P-treated HeLa/GFP-tubulin cells.

B[a]P-treatment resulted in binucleation and apoptosis in G1.

**Movie 5**. Fluorescence time-lapse microscopy of B[a]P-treated HeLa/GFP-tubulin cells.

B[a]P-treatment resulted in multipolar spindles and death in mitosis.

**Movie 6**. Fluorescence time-lapse microscopy of B[a]P-treated HeLa/GFP-tubulin cells.

B[a]P-treatment resulted in polylobed cell.

**Movie 7**. Fluorescence time-lapse microscopy of B[a]P-treated HeLa/GFP-tubulin cells.

B[a]P-treatment resulted in apoptosis in G1.

**Movie 8**. Fluorescence time-lapse microscopy of HeLa/GFP-centrin 2 cells in G2. Images were

captured every 3 min and displayed at 10 frames/s.

**Movie 9**. Fluorescence time-lapse microscopy of B[a]P-treated HeLa/GFP-centrin 2 cells.

B[a]P-treatment resulted in precocious centriole disengagement.
