# Supplementary material for: Hornerin mediates phosphorylation of the polo-box domain in Plk1 by Chk1 to induce death in mitosis
Source: Cell Death Differ. 2023 Aug 18;30(9):2151–66. doi: 10.1038/s41418-023-01208-y (PMC10482915; doi:10.1038/s41418-023-01208-y)

Figure 2C

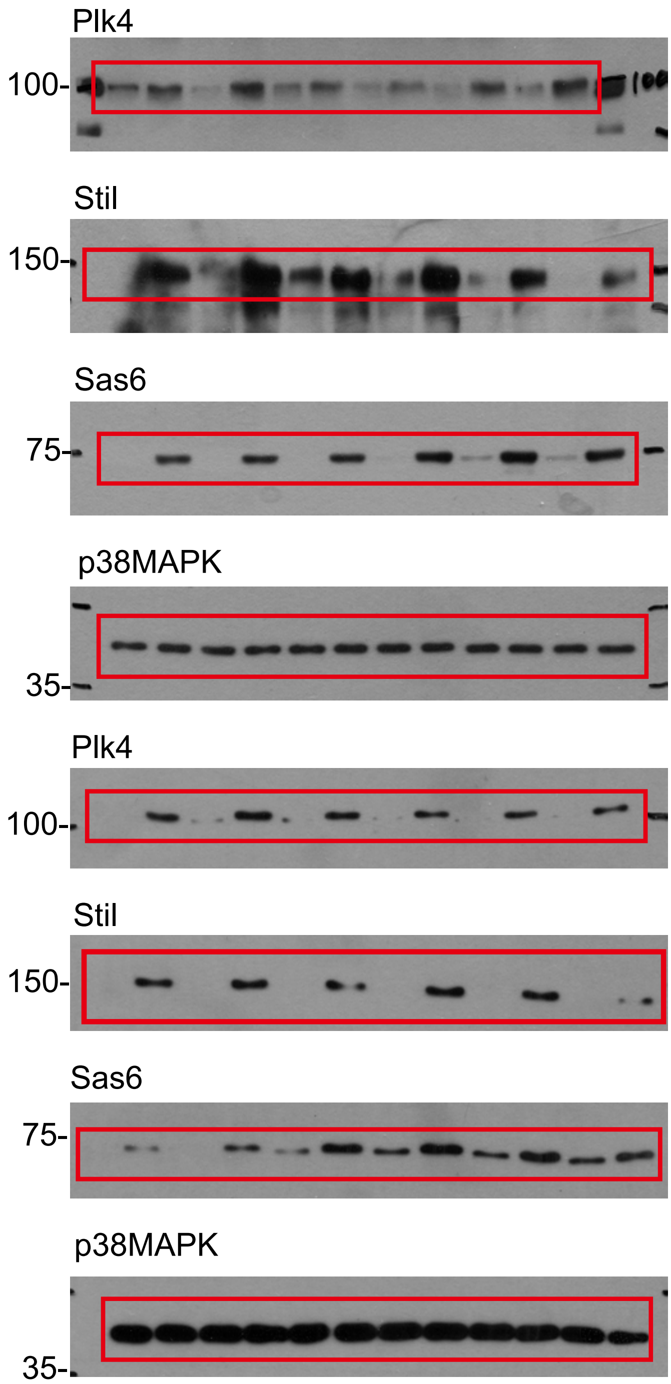

Figure 4A

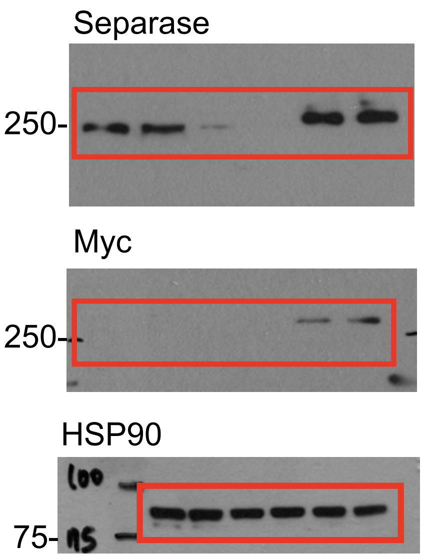

Figure 4E

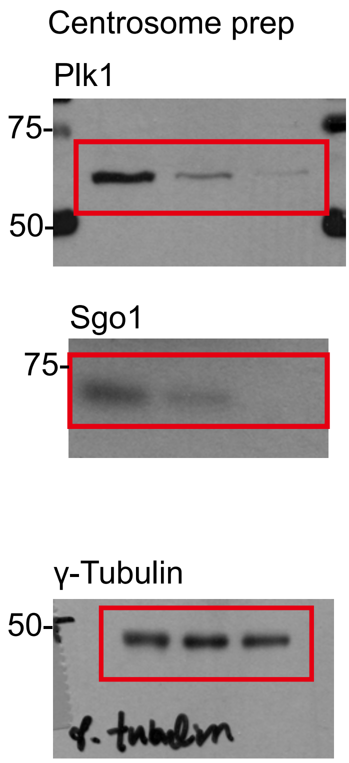

Figure 4F

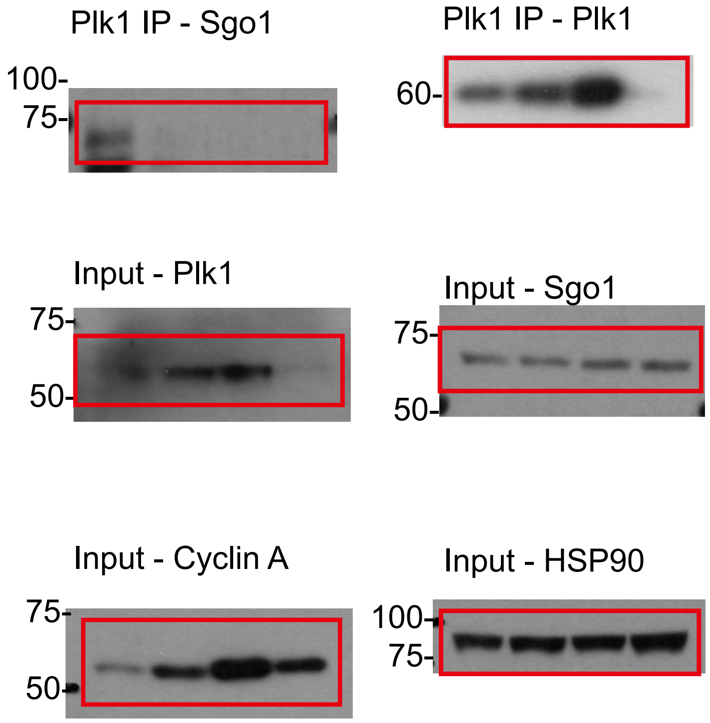

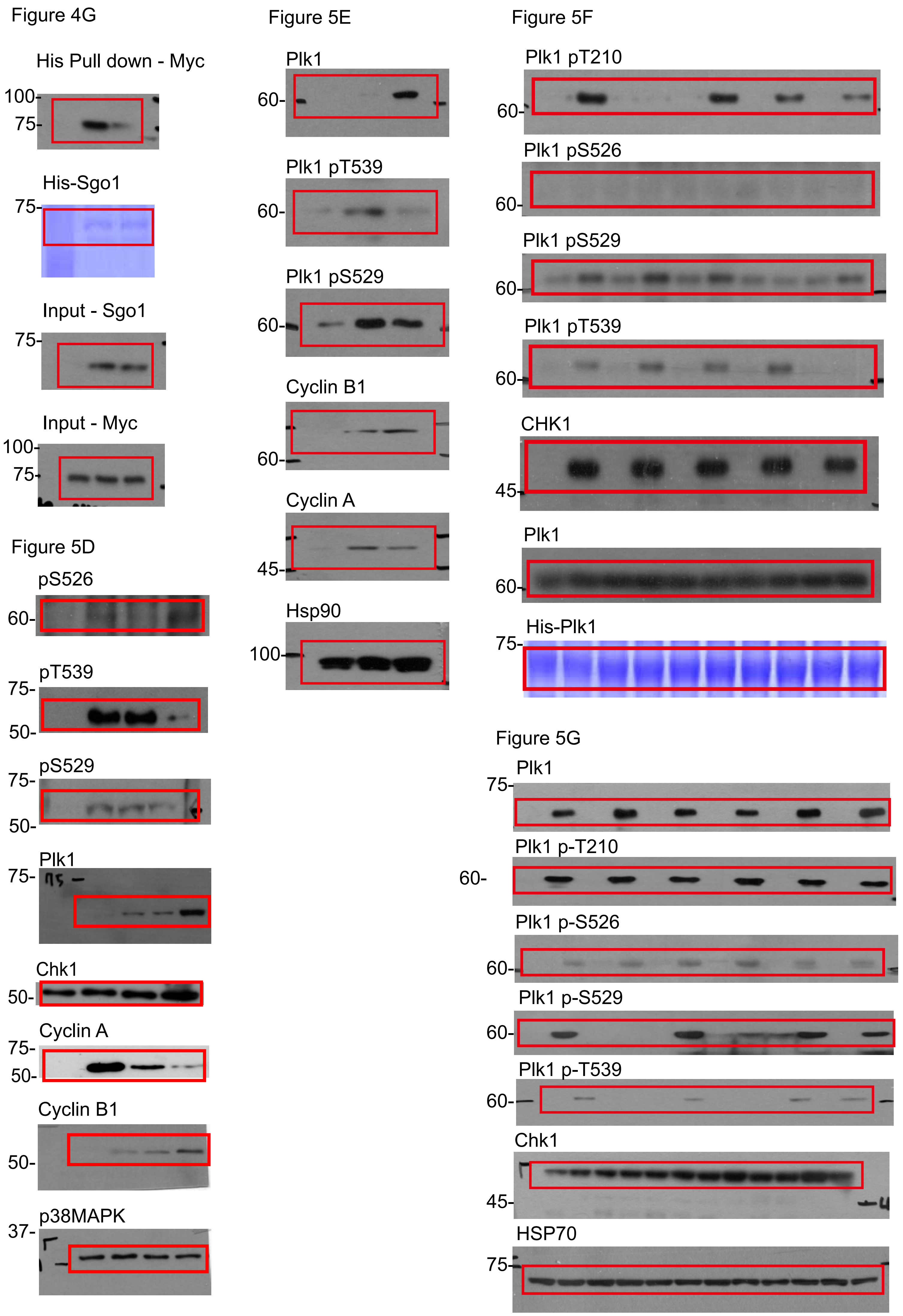
Figure 4G
His Pull down - Myc
100-
75-
His-Sgo1
75-
Input - Sgo1
75-
Input - Myc
100-
75-
Figure 5D
pS526
60-
pT539
75-
50-
pS529
75-
50-
Plk1
75-
Chk1
50-
Cyclin A
75-
50-
Cyclin B1
50-
p38MAPK
37-
Figure 5E
Plk1
60-
Plk1 pT539
60-
Plk1 pS529
60-
Cyclin B1
60-
Cyclin A
45-
Hsp90
100-
Figure 5F
Plk1 pT210
60-
Plk1 pS526
60-
Plk1 pS529
60-
Plk1 pT539
60-
CHK1
45-
Plk1
60-
His-Plk1
75-
Figure 5G
Plk1
75-
Plk1 p-T210
60-
Plk1 p-S526
60-
Plk1 p-S529
60-
Plk1 p-T539
60-
Chk1
45-
HSP70
75-

Figure 5H

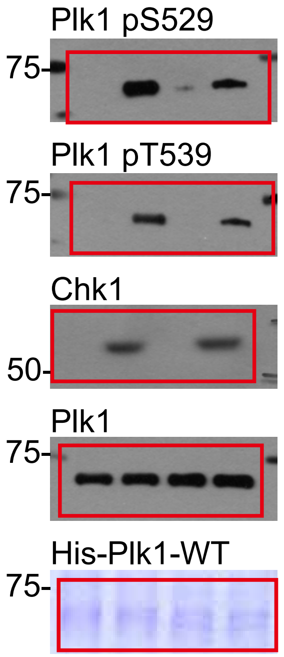

Figure 5I

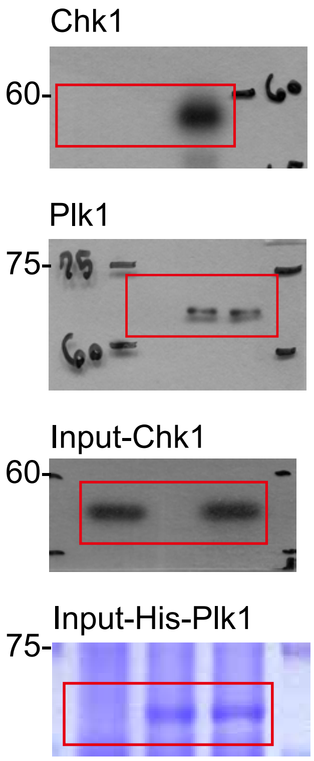

Figure 5J

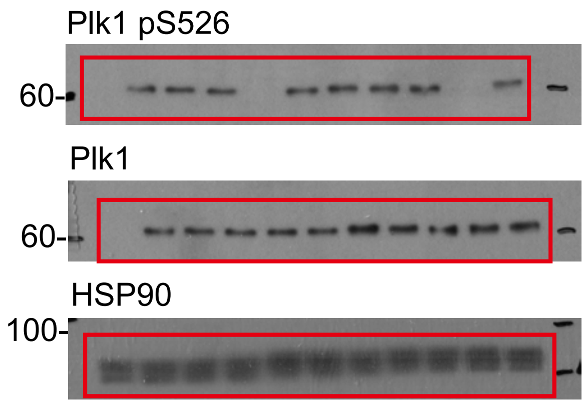

Figure 5K

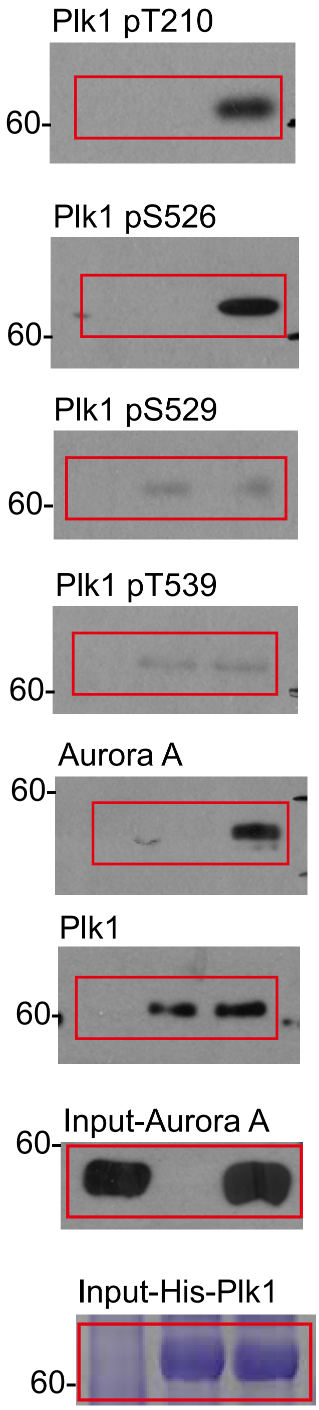

Figure 5L

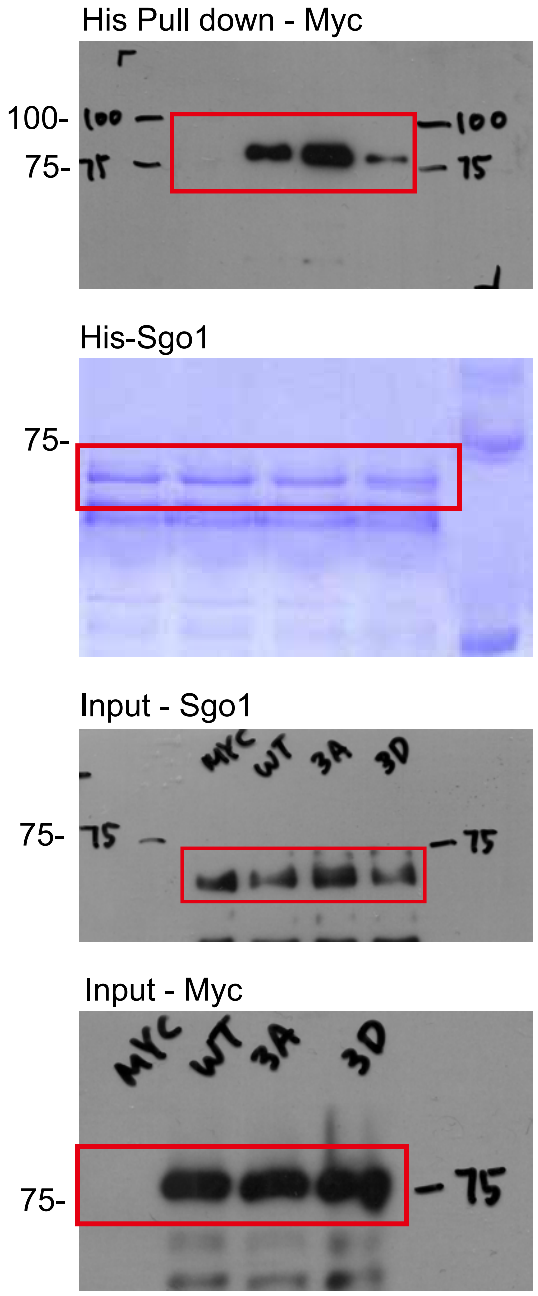

Figure 6A

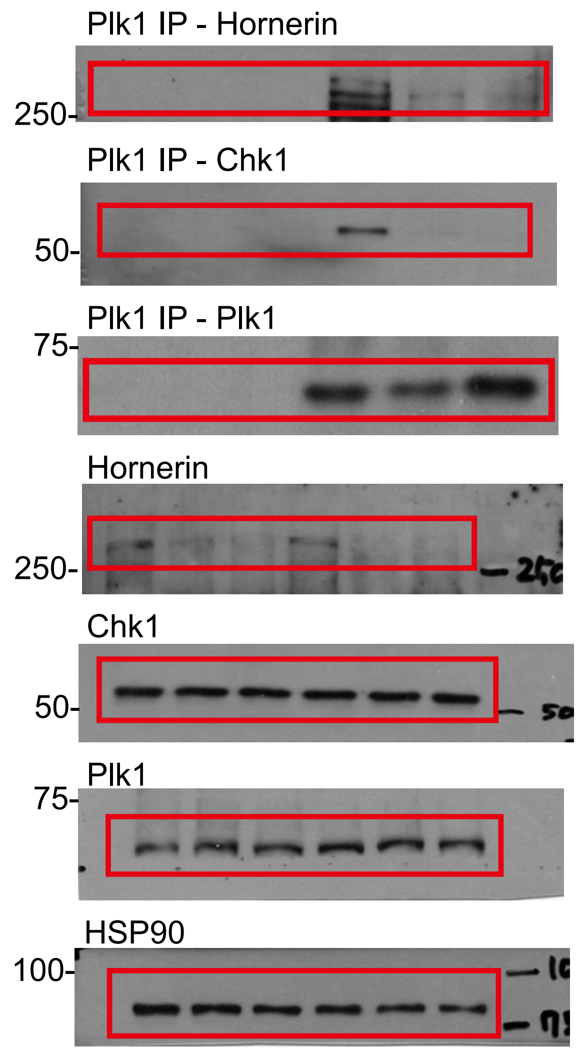

Figure 6D

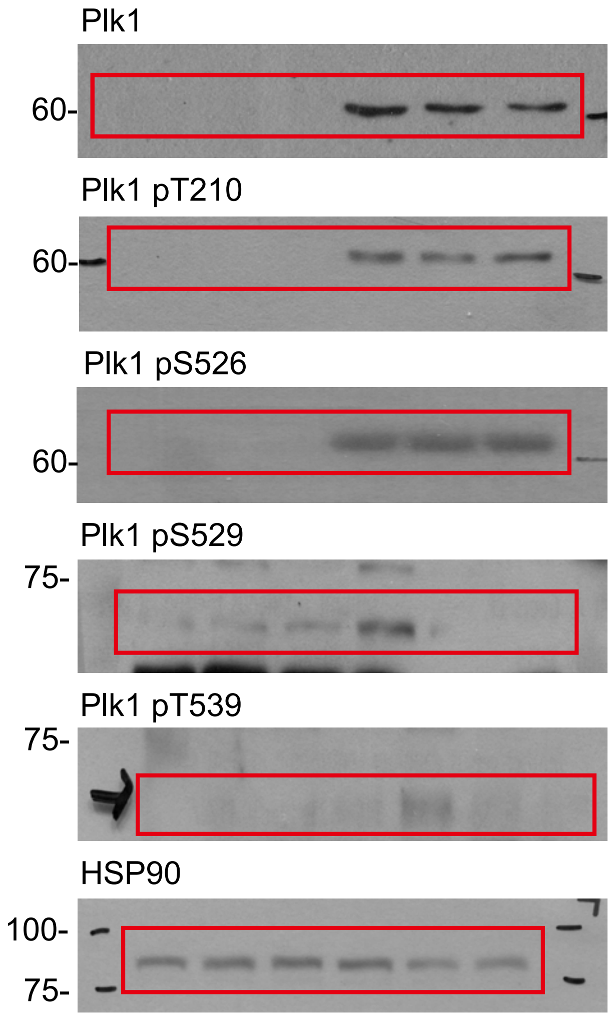

Figure 6E

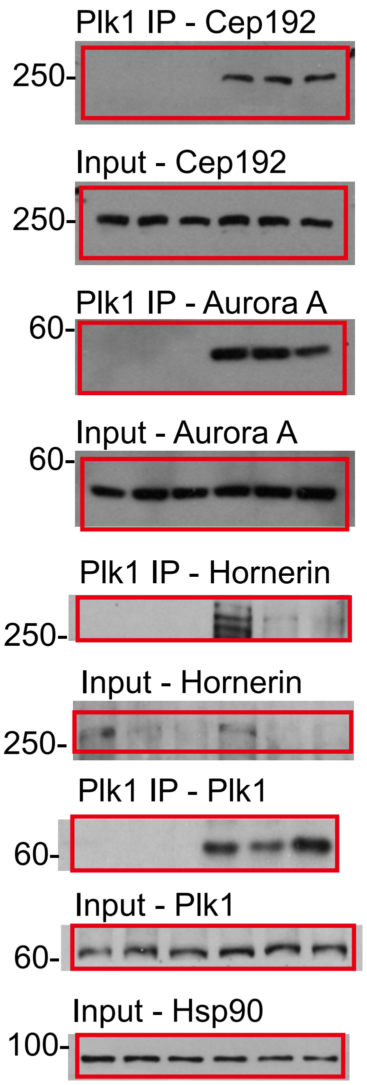

Figure 6F

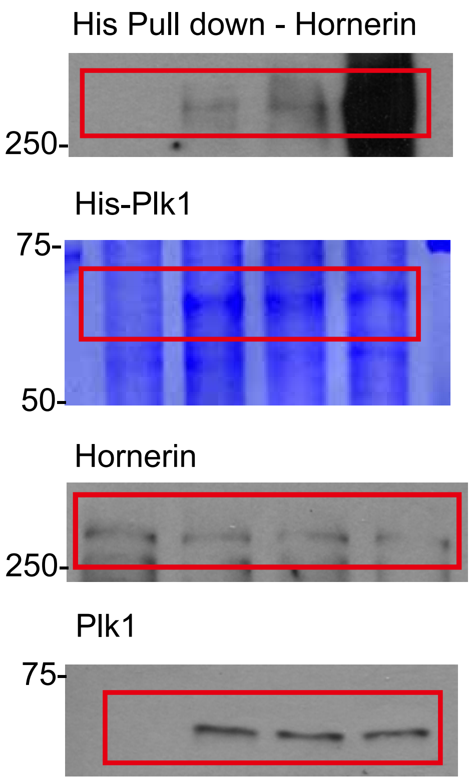

Figure 6G

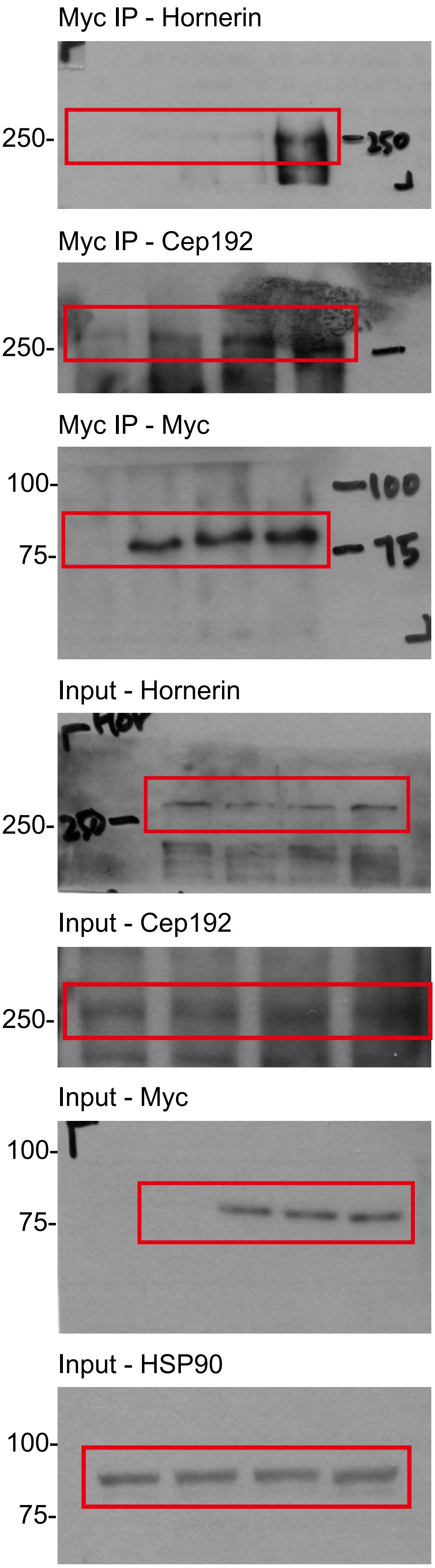

Figure 7H

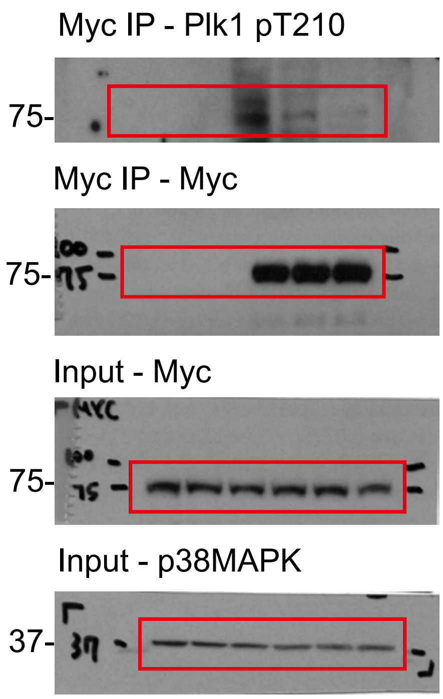

Figure 8E

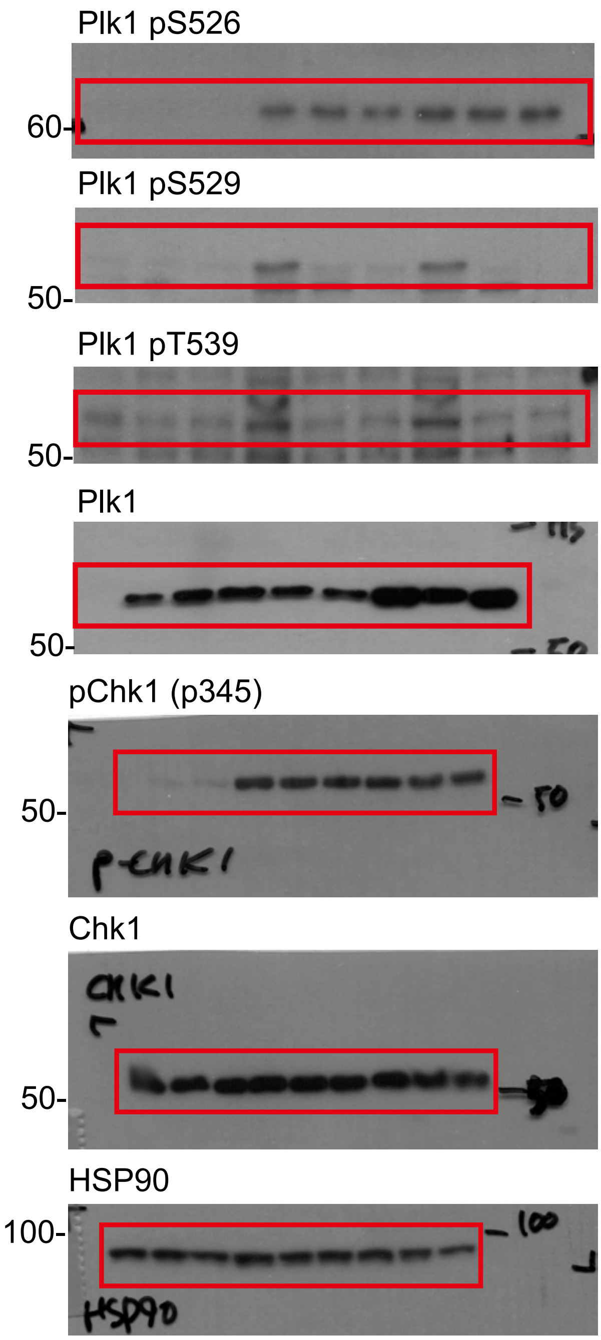

Figure S4C

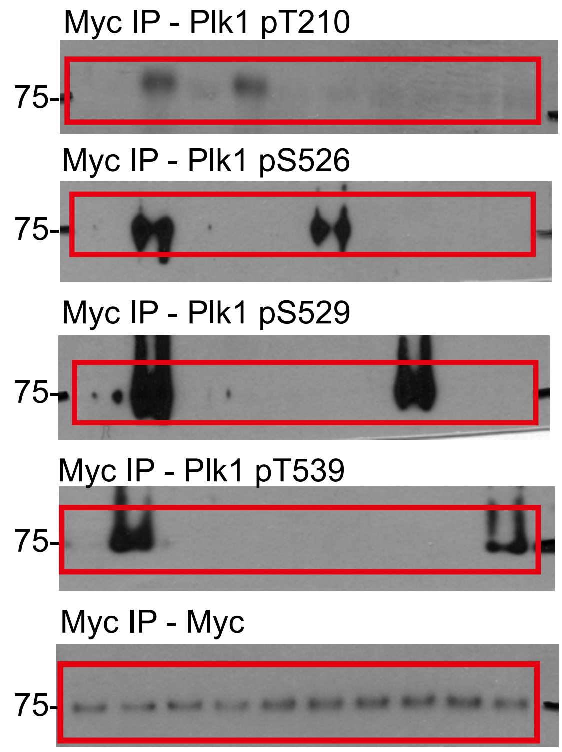

Figure S5B

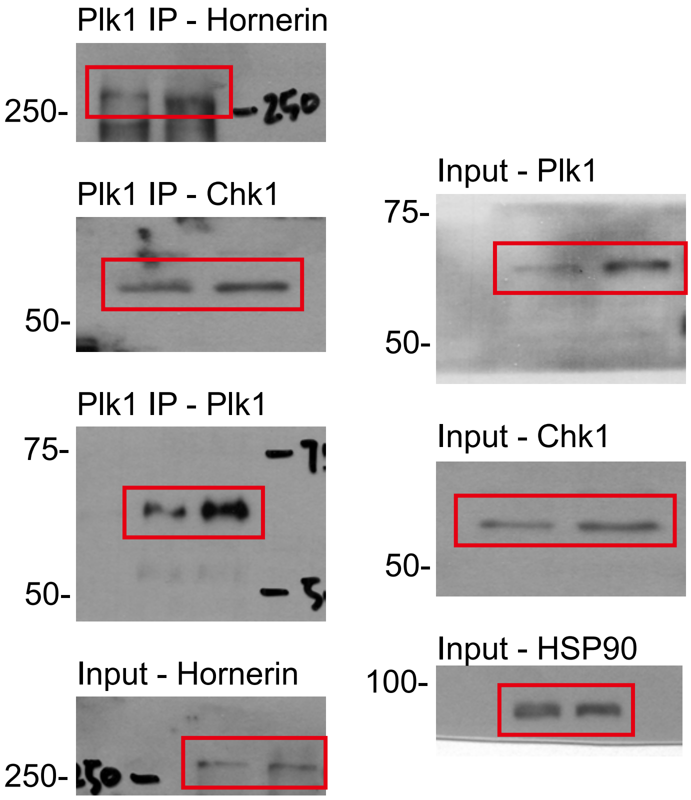

Figure S2

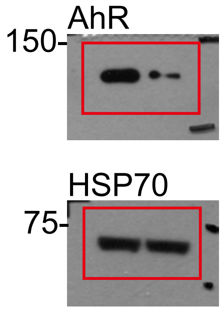

Figure S5D

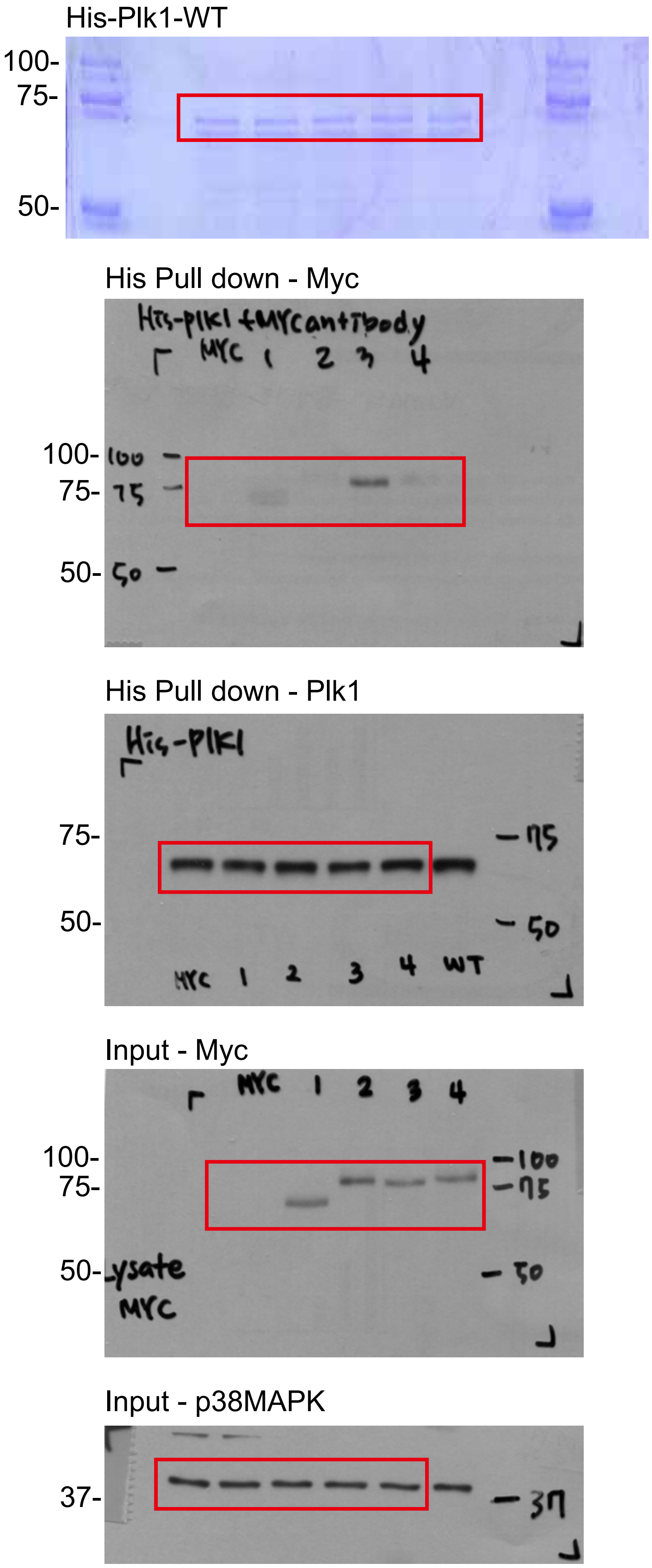

Figure S5E

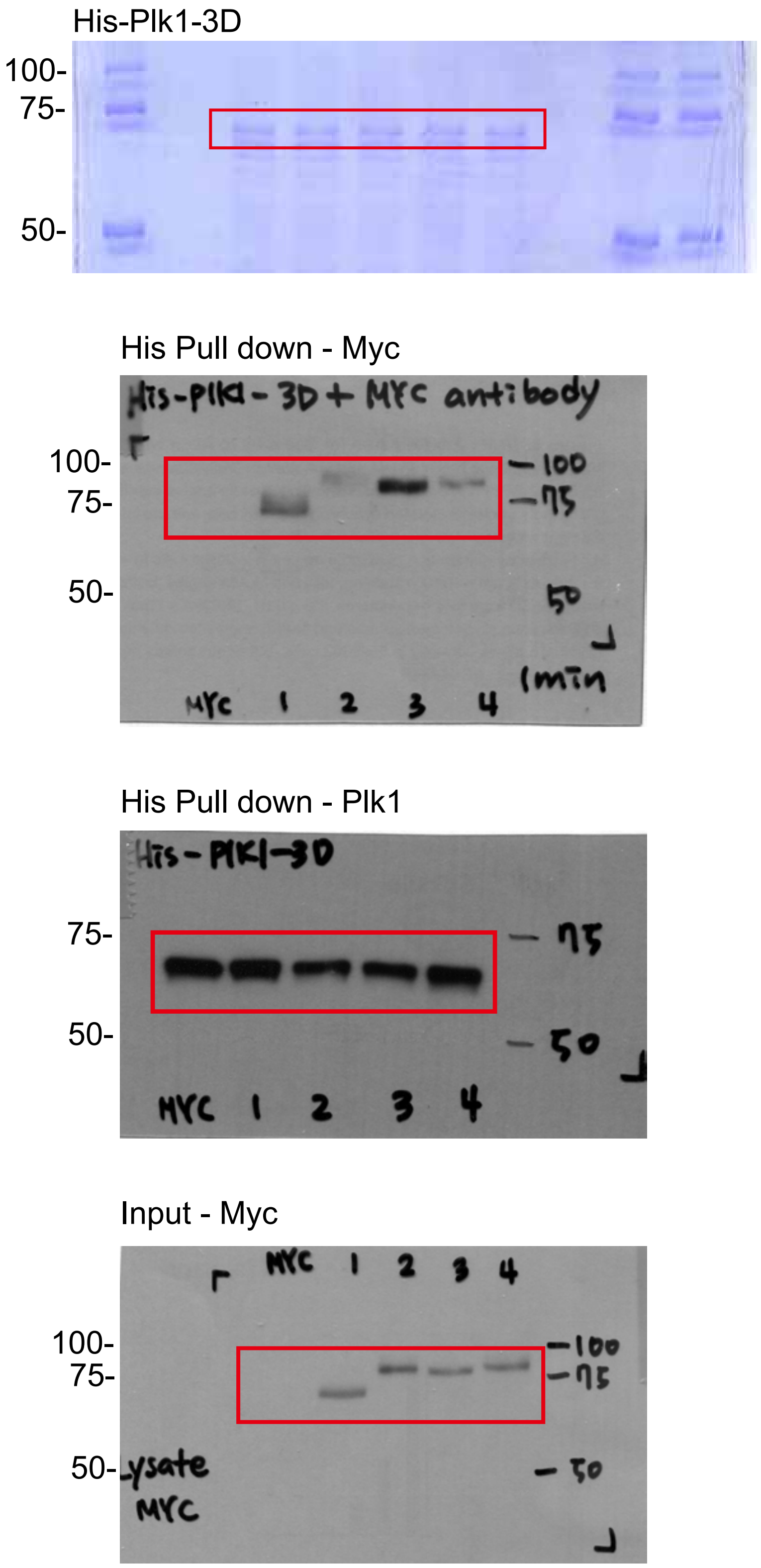

Figure S5F

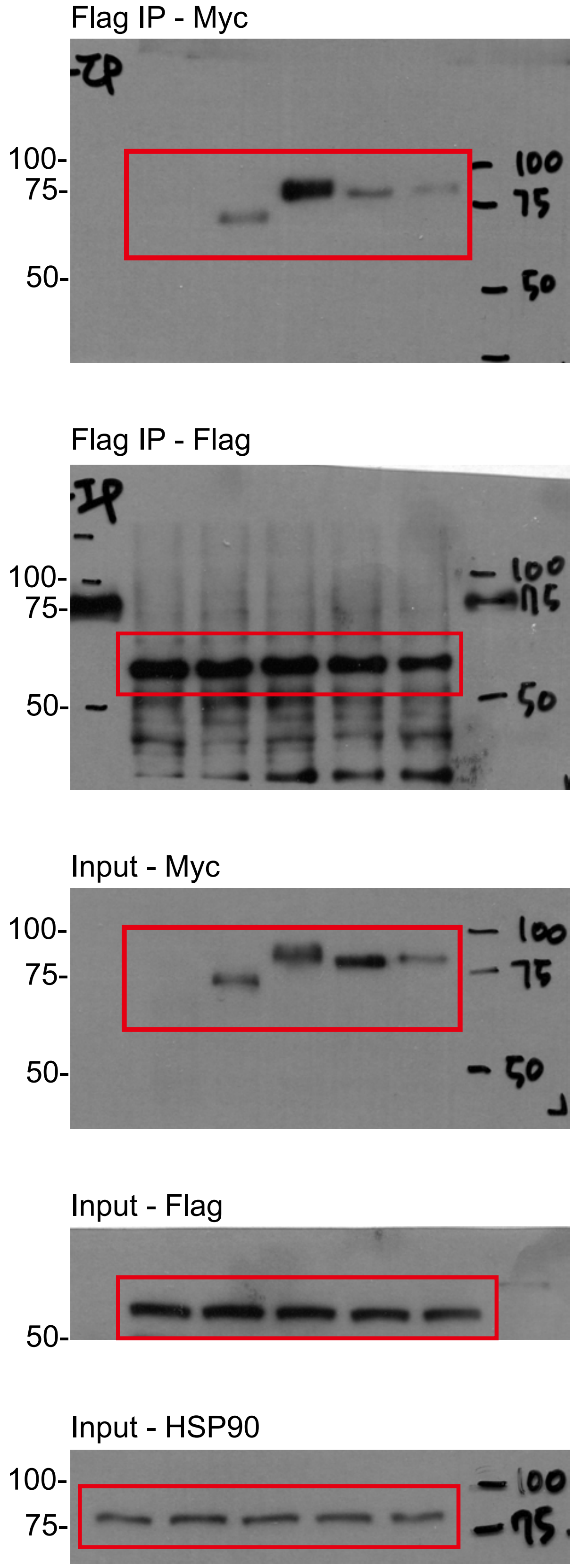

Figure S6C

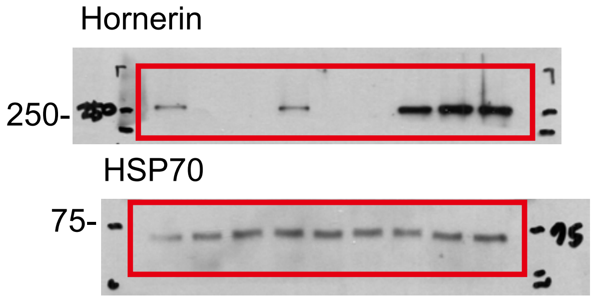

Figure S6E

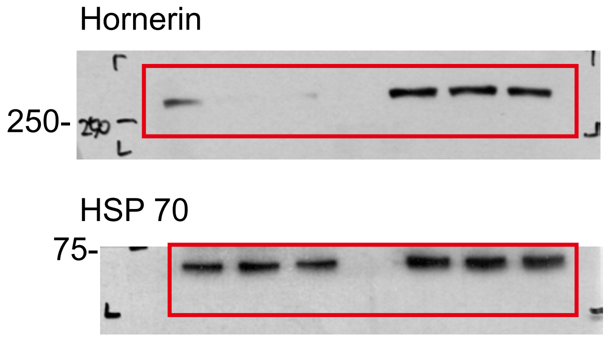

Figure S8A

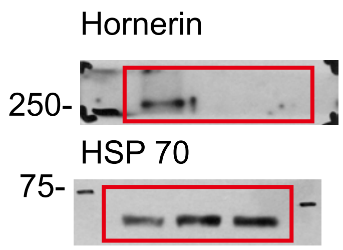

Figure S8B

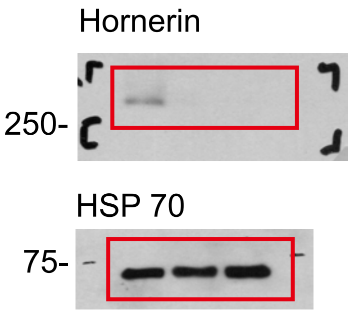

Figure S8C

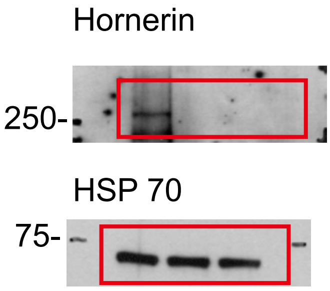

Figure S10B

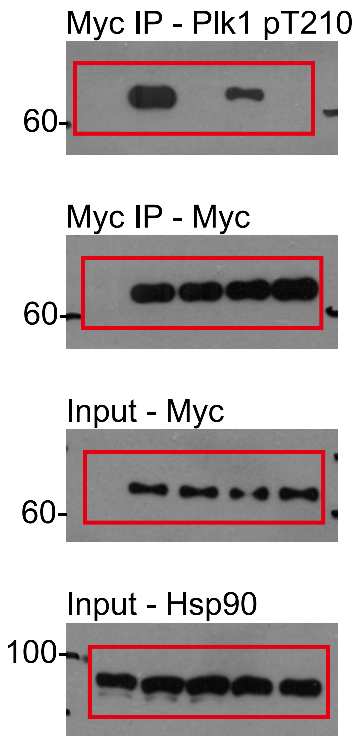

Supplement: Supplementary file 3 — Original Data File [file 41418_2023_1208_MOESM3_ESM.pdf]
